# *In Vitro* Pre-Clinical Validation of Suicide Gene Modified Anti-CD33 Redirected Chimeric Antigen Receptor T-Cells for Acute Myeloid Leukemia

**DOI:** 10.1371/journal.pone.0166891

**Published:** 2016-12-01

**Authors:** Kentaro Minagawa, Muhammad O. Jamil, Mustafa AL-Obaidi, Larisa Pereboeva, Donna Salzman, Harry P. Erba, Lawrence S. Lamb, Ravi Bhatia, Shin Mineishi, Antonio Di Stasi

**Affiliations:** Hematology/Oncology, University of Alabama at Birmingham, Birmingham, AL, United States of America; B.C. Cancer Agency, CANADA

## Abstract

**Background:**

Approximately fifty percent of patients with acute myeloid leukemia can be cured with current therapeutic strategies which include, standard dose chemotherapy for patients at standard risk of relapse as assessed by cytogenetic and molecular analysis, or high-dose chemotherapy with allogeneic hematopoietic stem cell transplant for high-risk patients. Despite allogeneic hematopoietic stem cell transplant about 25% of patients still succumb to disease relapse, therefore, novel strategies are needed to improve the outcome of patients with acute myeloid leukemia.

**Methods and findings:**

We developed an immunotherapeutic strategy targeting the CD33 myeloid antigen, expressed in ~ 85–90% of patients with acute myeloid leukemia, using chimeric antigen receptor redirected T-cells. Considering that administration of CAR T-cells has been associated with cytokine release syndrome and other potential off-tumor effects in patients, safety measures were here investigated and reported. We genetically modified human activated T-cells from healthy donors or patients with acute myeloid leukemia with retroviral supernatant encoding the inducible Caspase9 suicide gene, a ΔCD19 selectable marker, and a humanized third generation chimeric antigen receptor recognizing human CD33. ΔCD19 selected inducible Caspase9-CAR.CD33 T-cells had a 75±3.8% (average ± standard error of the mean) chimeric antigen receptor expression, were able to specifically lyse CD33^+^ targets *in vitro*, including freshly isolated leukemic blasts from patients, produce significant amount of tumor-necrosis-factor-alpha and interferon-gamma, express the CD107a degranulation marker, and proliferate upon antigen specific stimulation. Challenging ΔCD19 selected inducible Caspase9-CAR.CD33 T-cells with programmed-death-ligand-1 enriched leukemia blasts resulted in significant killing like observed for the programmed-death-ligand-1 negative leukemic blasts fraction. Since the administration of 10 nanomolar of a non-therapeutic dimerizer to activate the suicide gene resulted in the elimination of only 76.4±2.0% gene modified cells *in vitro*, we found that co-administration of the dimerizer with either the BCL-2 inhibitor ABT-199, the pan-BCL inhibitor ABT-737, or mafosfamide, resulted in an additive effect up to complete cell elimination.

**Conclusions:**

This strategy could be investigated for the safety of CAR T-cell applications, and targeting CD33 could be used as a ‘bridge” therapy for patients coming to allogeneic hematopoietic stem cell transplant, as anti-leukemia activity from infusing CAR.CD33 T-cells has been demonstrated in an ongoing clinical trial. Albeit never performed in the clinical setting, our future plan is to investigate the utility of iC9-CAR.CD33 T-cells as part of the conditioning therapy for an allogeneic hematopoietic stem cell transplant for acute myeloid leukemia, together with other myelosuppressive agents, whilst the activation of the inducible Caspase9 suicide gene would grant elimination of the infused gene modified T-cells prior to stem cell infusion to reduce the risk of engraftment failure as the CD33 is also expressed on a proportion of the donor stem cell graft.

## Introduction

Approximately fifty percent of patients with acute myeloid leukemia (AML) can be cured with current therapeutic strategies which include, standard dose chemotherapy for patients at standard risk of relapse as assessed by cytogenetic and molecular analysis, or high-dose chemotherapy with allogeneic hematopoietic stem cell transplant (allo-HSCT) for high-risk patients. Despite allo-HSCT about 25% of patients still succumb to disease relapse, therefore, novel strategies are needed to improve the outcome of patients with AML.

One approach to decrease disease relapse is to increase the anti-tumor activity of T-cells by genetic redirection, endowing them with a transgenic T-cell receptor (TCR) or a chimeric antigen receptor (CAR) molecule targeting a specific tumor associated antigen. However, TCR redirected T-cells are HLA restricted, and TCR mispairing with the endogenous TCR could result in reduced avidity or unwanted specificities [1]. Alternatively, CARs represent a universal platform for immune-therapy because they are not HLA-restricted, combining the specificity of an antibody with the killing machinery of the T-cell in a single chain [2], with a minimized risk of chain mispairing. Additionally, recognizing antigens in an HLA independent fashion makes CAR T-cells intrinsically resistant to immune evasion strategies that could arise during antigen processing or presentation [3]. Infusion of CAR.CD19 redirected T-cells has resulted in complete remission/partial remission of lymphoid leukemia, but at the expense of severe cytokine release syndrome and other off-tumor effects [4]. CAR T-cell applications for AML have been investigated *in vitro* and in mice models [5] targeting CD33 [6–9], CD44v6 [10], CD123 [5, 9, 11, 12], but only results from small clinical trials targeting Lewis-Y (LeY) [13], or CD33 [14] have been published to date.

We generated a CAR molecule encoding a humanized anti-CD33 single chain variable fragment (scFv) for the genetic modification of human activated T-cells to target CD33^+^ AML. CD33 is a myeloid-specific sialic acid-binding receptor overexpressed on the cell surface of 90% of AML blasts, and it has a role in regulating leukocyte functions in inflammatory and immune responses [15]. CD33 is also expressed on multipotent myeloid precursors, but not all normal hematopoietic stem cells, unipotent colony forming cells, maturing granulocytes and monocytes, peripheral granulocytes and resident macrophages, Kupfer cells and hepatocytes [16, 17].

Therapeutic strategies targeting CD33 with unconjugated antibodies, antibody-drug conjugates, immunotoxins, or radioisotopes, (either monospecific or targeting multiple antigens), have been developed or investigated in the clinical setting, and has been reviewed elsewhere [18]. Unconjugated monospecific antibodies have demonstrated modest activity in AML, with the clinical challenge of the need for continuous intravenous administration in virtue of their short half-life. Gemtuzumab ozogamicin (GO), a humanized CD33 antibody conjugated to a calicheamicin-γ_1_ derivative via a hydrolyzable linker, demonstrated clinical activity when given with induction chemotherapy in newly diagnosed AML, with mixed results depending on disease subtype, cytogenetic risk, and patient age. To overcome some of the limitations of GO, such as the non-uniform conjugation of the toxin with the antibody, the drug’s relatively slow internalization kinetics, and toxin extrusion via drug transporters, SGN-CD33A, a humanized CD33 antibody with engineered cysteines carrying a synthetic DNA cross-linking pyrrolobenzodiazepine dimer via a protease-cleavable linker, was developed and demonstrated increased potency in vitro against human AML cells while maintaining activity in the presence of drug transporters. Complete remissions were seen in 30% of patients in an ongoing phase 1 study of primarily older adults with relapsed/refractory AML, or those who declined standard intensive therapy for newly diagnosed disease (NCT01902329). CAR T-cells present several advantages over the infusion of therapeutic antibody conjugates, such as the more efficient bio-distribution and persistence, and independence from the multidrug resistance protein. It is unclear whether targeting CD33 with a CAR would result in hepatic toxicity as seen with GO [19, 20], however, considering that administration of CAR T-cells has been associated with cytokine release syndrome and other potential off-tumor effects in patients [4], safety measures are here investigated.

To enable elimination of the CAR T-cells in case of severe adverse events (SAEs), we incorporated the intracellular inducible Caspase9 (iC9) suicide gene, composed of a drug binding domain cloned in frame with human Caspase9, with the exogenous administration of a non therapeutic small molecule chemical inducer of dimerization (CID) (AP1903 *in vivo*, or the analogous AP-20187 for *in vitro* studies), resulting in iC9 dimerization and apoptosis of the transduced cells within hours. This has been clinically validated by our group [21–23], and an imminent phase 1 clinical trial will investigate iC9 and a CAR T-cells redirected against the disialoganglioside GD2 in patients with advanced melanoma (CARPETS, ACTRN12613000198729) [24].

The iC9 construct also includes a truncated (biologically inert) CD19 (ΔCD19) molecule, serving solely as a selectable marker. Here we validated the functionality of the ΔCD19 selected (sel.) iC9-CAR.CD33 ATCs *in vitro*, showing their activity against CD33^+^ AML cell lines or AML cells freshly isolated from patients. Additionally, since the activation of the iC9 suicide gene results in incomplete elimination (~90%) of gene modified cells even after higher doses or repeated administration [25, 26], we sought to investigate the addition of a second pharmacologic agent together with the dimerizer to enhance elimination.

We have previously demonstrated that iC9 resistant cells have higher expression of the anti-apoptotic molecule B-cell lymphoma 2 (BCL-2) [25], and therefore we investigated whether the co-administration of the dimerizer with an anti-apoptotic small molecule inhibitor would result in complete suicide gene modified cell elimination. We also tested the activity of the lymphotoxic agent mafosfamide. We found that co-administration of ABT-199 [27], ABT-737 [27], or mafosfamide [28] with the dimerizer resulted in an additive killing effect on suicide gene modified cells by activating the iC9 safety switch, up to complete elimination of the gene modified cells [29].

This strategy could be investigated for the safety of CAR T-cell applications, and targeting CD33 could be used as a ‘bridge” therapy for patients coming to allogeneic hematopoietic stem cell transplant, as anti-leukemia activity from infusing CAR.CD33 T-cells has been demonstrated in an ongoing clinical trial. [14] Albeit never performed in the clinical setting, our future plan is to investigate the utility of iC9-CAR.CD33 T-cells as part of the conditioning therapy for an allo-HSCT for AML, together with other myelosuppressive agents, whilst the activation of the iC9 suicide gene would grant elimination of the infused gene modified T-cells prior to stem cell infusion to reduce the risk of engraftment failure as the CD33 is also expressed on a proportion of the donor stem cell graft.

## Results

### Genetic modification of activated T-cells from healthy donors

**Fig 1:**
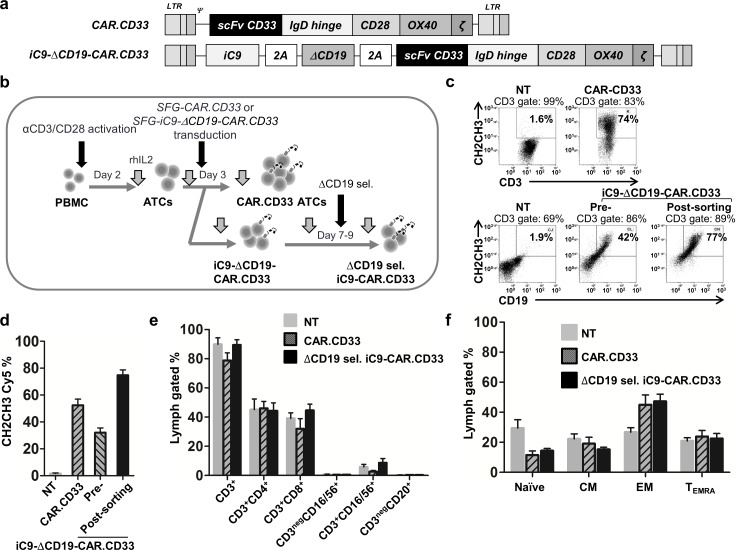
Generation and genetic modification of human activated T-cells. (a) Diagram of CAR.CD33, and iC9-ΔCD19-CAR.CD33 plasmid constructs used for the genetic modification of human activated T-cells (ATCs). (b) Schematic representation of the protocol for ATCs generation and transduction. (c) Representative experiment displaying the CAR expression on the surface of ATCs generated from a healthy donor. (d) CAR expression on non-transduced (NT), CAR.CD33, or iC9-ΔCD19-CAR.CD33 transduced ATCs assessed on day 11 after transduction, and on ΔCD19 selected (sel.) iC9-CAR.CD33 assessed on day 28 after transduction. (e) Lymphocyte subset marker’s expression on NT, CAR.CD33, or ΔCD19 sel. iC9-CAR.CD33 ATCs assessed by flow cytometry on day 11 after transduction. (f) Memory marker’s expression on NT, CAR.CD33, and ΔCD19 sel. iC9-CAR.CD33 ATCs (assessed on day 11, 11, or 28 after transduction, respectively); (mean± standard error of the mean (SEM); N: 4–7 experiments from 5 healthy donors for all the experiments). LTR: long terminal repeat; Ψ: packaging signal; scFv: single chain variable fragment; ζ: Zeta chain of the T-cell receptor; PBMC: peripheral blood mononuclear cells; CM: central memory; EM: effector memory; T_EMRA:_ terminal differentiated effector memory.

Activated T-cells (ATCs) were established from five healthy donors and transduced with retroviral supernatant encoding the CAR.CD33 or the iC9-ΔCD19-CAR.CD33 construct, (“Fig 1A and 1B”). As a control, we cultured non-transduced (NT) ATCs in parallel. As shown in “Fig 1C and 1D”, T-cells transduced with either the CAR.CD33 or the iC9-ΔCD19-CAR.CD33 construct expressed significant levels of CAR-CD33, although the expression was higher in cells transduced with the vector encoding CAR.CD33 alone (52±4.5% vs. 32±3.5%, respectively; average±standard error of the mean (SEM) is reported here and throughout the manuscript unless otherwise specified). However, after ΔCD19 selection of iC9-ΔCD19-CAR.CD33 ATCs, CAR expression was enriched to 75±3.8%. CAR.CD33 and ΔCD19 sel. iC9-CAR.CD33 gene modified ATCs expressed the CAR on both CD4 and CD8 subsets, and the majority of transduced T-cells (~70%) had a terminally differentiated/effector memory phenotype (N = 4–7 experiments from ATCs generated from 5 donors), (“Fig 1E and 1F”).

We evaluated the expression of the human CD33 antigen on freshly isolated peripheral blood mononuclear cells (PBMCs) or ATCs, and we found that 7.7±2.4% of PBMCs (N = 4; 3 donors), and 34±1.5% of NT ATCs (N = 4–5; 4 donors) also expressed the CD33 antigens; however, only 1.1±0.3% of ΔCD19 sel. iC9-CAR.CD33 ATCs expressed the CD33 antigen after three weeks in culture (N = 4–5; 4 donors, “Table A in S1 File”), and residual CD33 negative ATCs were able to expand *in vitro*. After three weeks in culture the fold expansion was 61±13 for NT, and 59±16 for ΔCD19 sel. iC9-CAR.CD33 ATCs, (N = 5–8, using ATCs from 4 donors, “Figure A in S1 File”).

### Effector functions

**Fig 2:**
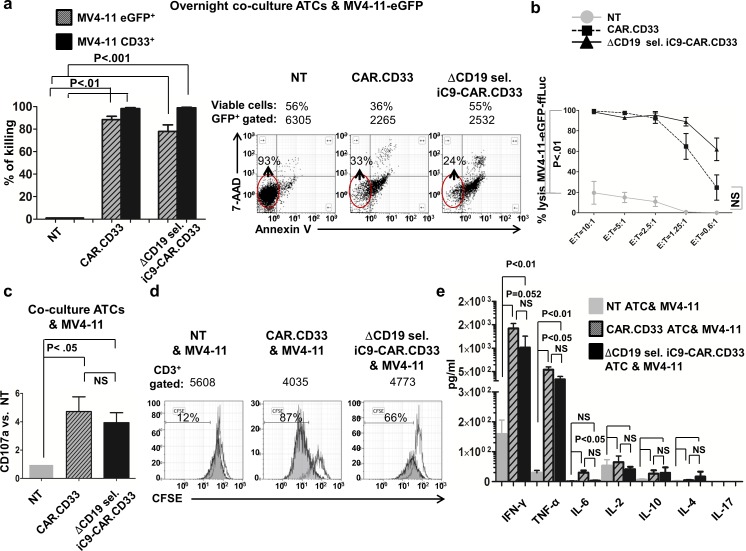
CAR.CD33, and ΔCD19 sel. iC9-CAR.CD33 ATCs displayed effectors function after stimulation with the CD33^+^ acute myeloid leukemia cell line, MV4-11. (a) Overnight co-culture experiments using non transduced (NT), CAR.CD33, or ΔCD19 sel. iC9-CAR.CD33 activated T-cells (ATCs) against the MV 4-11eGFP^+^ CD33^+^ cell line, followed by evaluation of enhanced green fluorescent protein (eGFP)/CD33^+^ cell killing by flow cytometry, as compared with co-culture employing NT ATCs as effectors; (mean±SEM of 3 experiments using ATCs from 3 healthy donors) *(left)*. Annexin V and 7-AAD expression from one representative co-culture experiment *(right)*. (b) Luciferase-based cytotoxicity assay using NT, CAR.CD33, or ΔCD19 sel. iC9-CAR.CD33 ATCs after overnight incubation with the CD33^+^ MV4-11 cell line engineered to express the eGFP-firefly-luciferase (eGFP-ffLuc) construct. Effector:target (E:T) ratios ranged from 10:1 to 0.6:1; (N: 3–7 experiments with ATCs generated from 5 healthy donors). (c) Evaluation of CD107a degranulation marker expression on CAR.CD33 or ΔCD19 sel. iC9-CAR.CD33 ATCs (as compared with NT ATCs) after 4 hours of stimulation with MV4-11 CD33^+^ targets (E:T = 4:1); (N: mean±SEM of 4–6 experiments with ATCs from 4 healthy donors). (d) CFSE-based proliferation assay stimulating NT, CAR.CD33, or ΔCD19 sel. iC9-CAR.CD33 ATCs with irradiated (40 Gy) MV4-11 CD33^+^ cell line *(gray)*, or no target cells *(blank)*, and cultured for additional 5 days (E:T = 1:1). Data from one representative experiment is shown. (e) Cytokines production on supernatant collected after overnight incubation of NT, CAR.CD33, or ΔCD19 sel. iC9-CAR.CD33 ATCs with the MV4-11 CD33^+^ cell line (E:T = 4:1); (mean±SEM of 3–9 independent experiments from 3–4 different healthy donors). SEM: standard error of the mean; NS: not statistically significant.

In order to assess whether the expression of a CAR molecule targeting CD33, onto CAR.CD33 ATCs or ΔCD19 sel. iC9-CAR.CD33 ATCs, made them cytotoxic to CD33^+^ tumor cells, we investigated their function in an *in vitro* co-culture assay, and a luciferase-reporter cytotoxicity assay. For co-culture assay, we used as a target the MV4-11 CD33^+^ AML cell line genetically modified to express an enhanced green fluorescent (eGFP) protein, and sorted by fluorescence-activated cell sorting (FACS) to eGFP expression ≥98%. We then co-cultured the MV4-11 eGFP^+^ cell line overnight with NT, CAR.CD33 or ΔCD19 sel. iC9-CAR.CD33 ATCs at an effector:target ratio of 4:1, without exogenous cytokines administration. Co-cultures employing CAR.CD33 ATCs resulted in 88.4±3.0% reduction of MV4-11 eGFP^+^ target cells, while co-cultures employing ΔCD19 sel. iC9-CAR.CD33 ATCs resulted in 78±6.0% reduction of MV4-11 eGFP^+^ target cells as compared with co-cultures employing NT ATCs as effectors, (N:3 from 3 donors; *P*<0.01 (CAR.CD33), and *P*<0.001 (ΔCD19 sel. iC9-CAR.CD33) vs. NT ATCs, respectively), (“Fig 2A”). In addition, by gating on the eGFP^+^ target cells, we estimated that co-cultures employing CAR.CD33 ATCs resulted in 98.2±0.9% reduction of CD33 expressing targets, and co-cultures employing ΔCD19 sel. iC9-CAR.CD33 ATCs resulted in 99±0.6% reduction of CD33 expressing targets, as compared with co-cultures employing NT ATCs, (N:3 from 3 donors; *P*<0.01 (CAR.CD33), and *P*<0.001 (ΔCD19 sel. iC9-CAR.CD33) vs. NT ATCs, respectively), (“Fig 2A”). The reduction of CD33^+^ cells was due to cell killing, and not to antigen downregulation, as demonstrated by Annexin V/7-amino-actinomycin D (7-AAD) staining and FACS analysis performed 4 hours after the beginning of the co-culture. The percentage of viable cells, as compared with NT, was 29±7.1% for CAR.CD33, or 15±2.9% for ΔCD19 sel. iC9-CAR.CD33 ATCs (N:3 from 3 donors; *P*<0.01 vs. NT ATCs for both). Dot-plots from a representative experiment are shown in “Fig 2A”, whereas a summary of the results is represented in the “Figure B in S1 File”. We also assessed the ability of CAR redirected ATCs to kill CD33^+^ targets in an *in vitro* firefly-luciferase-based cytotoxicity assay. After overnight incubation with MV4-11 *eGFP-Firefly Luciferase*^+^ *(ffLuc)* target cells at an effector:target ratio of 10:1, we observed 99.3±0.4% killing when using CAR.CD33 ATCs as effectors, and 98.3±0.5% killing when using ΔCD19 sel. iC9-CAR.CD33 ATCs as effectors, (N: 3–7 from 5 donors; *P*<0.05, and *P*<0.001 vs. NT ATCs, respectively); significant killing was maintained until the 1.25:1 ratio, and a summary of the results is depicted in “Fig 2B”.

Next, we assessed the ability of CAR redirected ATCs to release the lysosomal-associated membrane protein-1 (LAMP-1 or CD107a), a marker of CD8^+^ T-cell degranulation. For this aim, following stimulation with CD33^+^ targets, we performed intracellular cytokine staining with a CD107a antibody. We found that CAR.CD33 expressed CD107a at a 4.7±1.0 folds higher than NT ATCs, and ΔCD19 sel. iC9-CAR.CD33 expressed CD107a at a 3.9±0.7 folds higher than NT ATCs, (N:4–6 from 4 donors; *P*<0.05 vs. NT ATCs for both), (“Fig 2C”).

To evaluate the proliferative response of CAR redirected ATCs in response to CD33^+^ target stimulation *in vitro*, we co-cultured effectors with irradiated MV4-11 cell line at an effector:target ratio of 1:1 for 5 days, without exogenous cytokines administration. CAR redirected ATCs exhibited a dilution of the carboxyfluorescein succinimidyl ester (CFSE) dye at a higher extent as compared with NT, as per proliferation in response to CD33^+^ target, (“Fig 2D”).

In addition, to confirm that stimulation of CAR redirected ATCs with CD33^+^ targets would result in secretion of Th1 cytokines, we measured Th1/Th2/Th17 cytokines in the supernatant after co-culture with the CD33^+^ MV4-11 cell line. CAR redirected ATCs had a robust interferon-gamma (IFN-γ) secretion (NT: 159±47, CAR.CD33: 1,423±103, or ΔCD19 sel. iC9-CAR.CD33: 1,012±242 pg/mL; *P* = 0.052, and *P*<0.01 vs. NT ATCs, respectively), and tumor necrosis factor-alpha (TNF-α) production (NT: 30±7.9, CAR.CD33: 540±61, or ΔCD19 sel. iC9-CAR.CD33: 332±64 pg/mL; *P*<0.05, and *P*<0.01 vs. NT ATCs, respectively). For each respective conditions as per above, other cytokines were detected as follows: interleukin-6 (IL-6) production (1.1±0.3; 30±7, 2.9±1.3 pg/mL; *P*<0.05 vs. NT ATCs for CAR.CD33. ATCs), IL-2 (53±20, 65±21, 42±8.2 pg/mL), IL-10 (6.7±1.5, 27±11; 30±18 pg/mL), and IL-4 (1.3±1.3; 4.7±1.0;17±17 pg/mL). IL-17A production was below 5 pg/mL; (mean±SEM of 3–9 independent experiments from 3–4 different donors), (“Fig 2E”). Target cells only resulted in a cytokine production below 5 pg/mL for all tested cytokines. Therefore, CAR.CD33, and ΔCD19 sel. iC9-CAR.CD33 ATCs demonstrated potent effector functions towards CD33^+^ leukemia cell lines.

### CAR.CD33 and ΔCD19 sel. iC9-CAR.CD33 ATCs eliminated freshly isolated blasts *in vitro*

**Fig 3:**
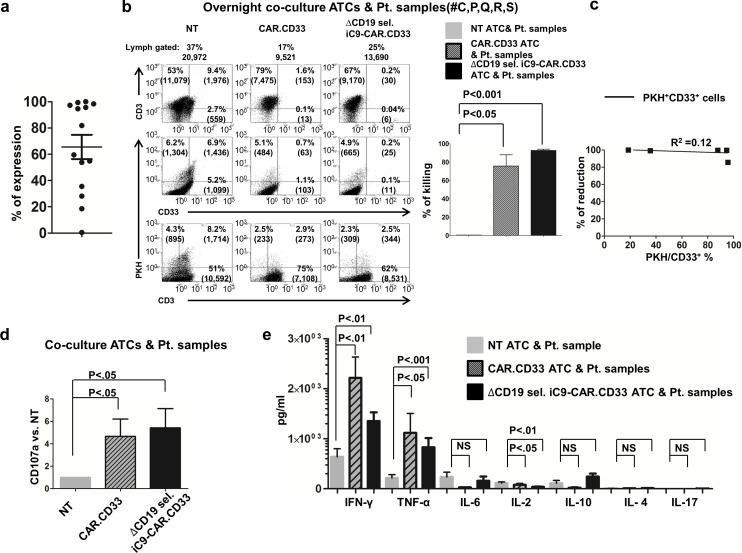
CAR.CD33, and ΔCD19 sel. iC9-CAR.CD33 ATCs exhibit anti-tumor activity against samples from patients with active AML. **(a)** CD33 expression on freshly isolated samples from patients with active acute myeloid leukemia (AML) evaluated by flow cytometry after gating on side scatter^low^/CD45^dim^ cells; (N:9 peripheral blood; N:5 bone marrow samples from different patients). (b) Overnight co-culture assay of non transduced (NT), CAR.CD33, or ΔCD19 sel. iC9-CAR.CD33 ATCs, generated from 3 healthy donors, with samples collected from patients with active AML labeled with the PKH-26 dye (effector:target (E:T) = 4:1), (patients#C, P, Q, R, and S). Results from a representative co-culture experiment (patient#C) gated on viable residual cells displaying the expression of *(i)* PKH vs.CD33, or *(ii)* PKH vs.CD3, or *(iii)* CD3 vs. CD33, *(left)*. Summary results from 3–9 independent experiments displaying average (±SEM) percentage of cell killing is shown, *(right)*. (c) Correlation (coefficient R^2^) between the reduction of PKH^+^CD33^+^ cells and CD33 expression level of the ΔCD19 sel. iC9-CAR.CD33 ATCs co-cultured with patient samples as represented in Fig 2B; (N: 9 independent experiments using ATCs from 3 healthy donors). (d) Evaluation of CD107a expression on CAR.CD33 or ΔCD19 sel. iC9-CAR.CD33 ATCs (as compared with NT ATCs) assessed 4 hours after stimulating them with samples collected from patients with AML (effector:target (E:T) = 4:1); (N:8–12 experiments using ATCs from 4 healthy donors). (e) Cytokines production (picograms per mL: pg/mL) on supernatant collected after overnight incubation of NT, CAR.CD33, or ΔCD19 sel. iC9-CAR.CD33 ATCs generated from healthy donor with samples isolated from 8 patients with AML (#C, G, L, N, O, P, Q, R, S, and U) (E:T = 4:1); (mean±SEM from 5–18 experiments using ATCs generated from 4 different healthy donors). ATCs: activated T-cells; Pt(s): patient(s); lymph: lymphocyte; SEM: standard error of the mean; NS: not statistically significant.

We sought to investigate whether CAR.CD33 and ΔCD19 sel. iC9-CAR.CD33 ATCs would result in the killing of CD33^+^ leukemia blasts freshly isolated from the peripheral blood or the bone marrow of patients with AML. The percentage of CD33 expression analyzed on AML samples from 14 patients is displayed in “Fig 3A”, and reported in “Table 1”, with an average expression of 66±9.2%. We then investigated the cytotoxic function of CAR redirected ATCs generated from healthy donors in an *in vitro* co-culture assay by labeling patient samples (Patients #C, P, Q, R, S) with the PKH-26 dye, at an effector:target ratio of 4:1, in the absence of exogenous cytokines administration. Co-cultures employing CAR.CD33 ATCs as effectors resulted in 76±12% reduction of CD33^+^ PKH^+^ targets, and co-cultures employing ΔCD19 sel. iC9-CAR.CD33 ATCs as effectors resulted in 93±1.4% reduction of CD33^+^ PKH^+^ targets (N:3–9 independent experiments (3 ATCs donors and 5 patients (Patients#C, P, Q, R, and S); *P*<0.05 (CAR.CD33), and *P*<0.001 (ΔCD19 sel. iC9-CAR.CD33) vs. NT ATCs, respectively). Dot plots from a representative experiment (patient#C) are shown in “Fig 3B”. We performed linear regression analysis on the experiments represented in “Fig 3B”, and we found that both CD33^dim^ and CD33^bright^ targets were eliminated in PKH^+^CD3^neg^CD33^+^ AML blast cells (R^2^ = 0.12), (“Fig 3C”).

**Table 1:** CD33 expression on AML patient samples.

Patient#	BM/PB^a^	CD33% on AML^b^ blasts
C	BM	95.6
K	BM	0.42
L	BM	28.1
M	BM	53.8
N	BM	59.5
O	BM	54.9
P	BM	35.5
Q	PB	18.5
R	PB	94.6
S	BM	81.9
T	PB	100
U	PB	98.4
V	BM	97.3
3	PB	99.4

De-identified samples from 14 patients with AML were stained with CD33 antibody and analyzed by flow cytometry.

^a^BM/PB: bone marrow sample/ peripheral blood sample.

^b^AML: Acute myeloid leukemia.

Effector ATCs generated from healthy donors stimulated with samples from patients with AML *in vitro* for 4 hours showed an increased expression of the CD8^+^ T-cell degranulation marker CD107a, with a 4.7±1.5-fold increase for CAR.CD33 as compared with NT (N = 8), and a 5.4±1.7-fold increase for ΔCD19 sel. iC9-CAR.CD33 as compared with NT, (N:8–12 from 4 donors; *P*<0.05 for both), (“Fig 3D”). Co-culture employing CAR.CD33 or ΔCD19 sel. iC9-CAR.CD33 ATCs as effector resulted in a robust secretion of IFN-γ (NT: 641±160, CAR.CD33: 2,218±418, or ΔCD19 sel. iC9-CAR.CD33: 1,353±177 pg/mL; *P*<0.01 vs. NT ATCs for both CAR.CD33 and ΔCD19 sel. iC9-CAR.CD33 ATCs), and TNF-α (215±69; 1,120±385; 832±183 pg/mL; *P*<0.05, and *P*<0.001 vs. NT ATCs, respectively). For each respective condition noted above, other cytokines were detected as follows: IL-6 (237±94; 29±10; 162±84 pg/mL), IL-2 (116±20; 79±27; 38±5.8 pg/mL; *P*<0.05, and *P*<0.01 vs. NT, respectively), IL-10 (110±57; 21±15; 243±62 pg/mL), and IL-4 (6.7±3.2; 8.8±6.3; 13±4.7 pg/mL). IL-17A production was below 5 pg/mL in each co-culture; (mean±SEM from 5–18 experiments from 4 different ATC donors), (“Fig 3E”).

**Fig 4:**
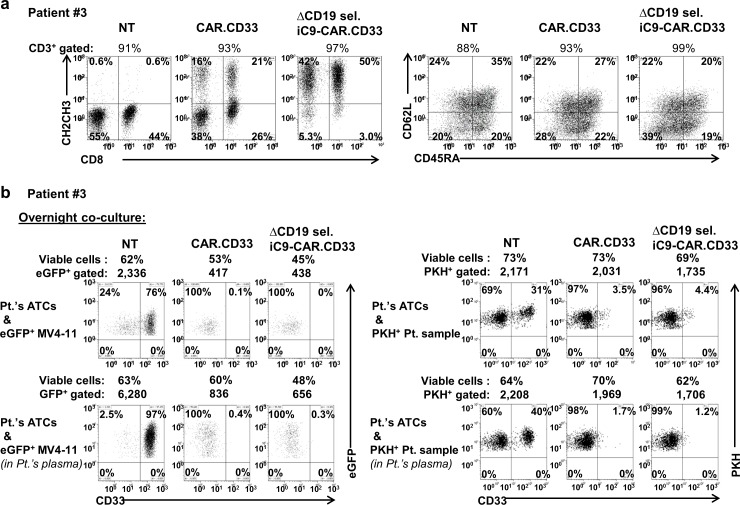
ΔCD19 sel. iC9-CAR.CD33 ATCs generated from a patient with AML. (a) Peripheral blood mononuclear cells isolated from a patient with acute myeloid leukemia (AML) (patient#3) were used to generate activated T-cells (ATCs). Non transduced (NT), CAR.CD33, or ΔCD19 sel. iC9-CAR.CD33 ATCs were stained with the appropriate monoclonal antibody, and analyzed by flow cytometry after gating on the CD3^+^ population to analyze CD8 and CAR expression, *(left)* or CD45RA and CD62L expression, *(right)*. (b) Dot plots from a representative co-culture experiment employing NT, CAR.CD33, *or* ΔCD19 sel. iC9-CAR.CD33 ATCs from patient#3 challenged with the MV4-11 CD33^+^ AML cell line genetically modified to express the enhanced green fluorescent protein (eGFP) marker *(left)*, or with an autologous CD33^+^ AML sample *(right*); the co-cultures were performed in the presence of medium *(top panels)* or in the presence of autologous patient’s plasma *(bottom panels)*.

In order to assess whether it would be feasible to translate this strategy into the clinical setting, we were able to successfully generate CAR.CD33 or ΔCD19 sel. iC9-CAR.CD33 ATCs using PBMC isolated from patients with AML. “Fig 4” shows the phenotypic characteristics and cytotoxic activity of NT, CAR.CD33 and ΔCD19 sel. iC9-CAR.CD33 ATCs generated from two patients with AML (patients#3, and #U). Fold expansions from ATC generated from these two patients after three weeks of culture were 100±66 for NT, 60±10 for CAR.CD33, and 66±16 for ΔCD19 sel. iC9-CAR.CD33 ATCs, (“Figure C in S1 File”). We tested the cytotoxic activity of ATCs generated from patient#3 after overnight incubation with the CD33^+^ MV4-11-eGFP^+^ cell line, in the presence of culture medium or in the presence of patient’s autologous plasma. ATCs generated from patient#3 proved functional, resulting in the elimination of CD33^+^ targets after overnight co-culture (CAR.CD33: 98±2.0%; or ΔCD19 sel. iC9-CAR.CD33: 98±2.5%, vs. NT ATCs), and the presence of patient’s plasma did not impair the killing activity of ATCs (CAR.CD33: 99±1.1%, or ΔCD19 sel. iC9-CAR.CD33: 100±0.07%, vs. NT ATCs), (“Fig 4B”, left panels). ATCs generated from patient#3 resulted also in the elimination of PKH^+^ labeled CD33^+^ autologous targets (CAR.CD33: 89±0.6%; or ΔCD19 sel. iC9-CAR.CD33: 89±0.004%, vs. NT ATCs), and the presence of patient’s plasma did not impair the killing activity of ATCs (CAR.CD33: 97±1.1%, or ΔCD19 sel. iC9-CAR.CD33: 98±0.6%, vs. NT ATCs), (“Fig 4B”, right panels). ATCs derived from patient#U were also functional (“Figure D in S1 File”). Cell elimination was due to apoptosis as assessed by Annexin V/7-AAD staining performed 4 hours from the beginning of the co-culture (“Figure E in S1 File”).

**Fig 5:**
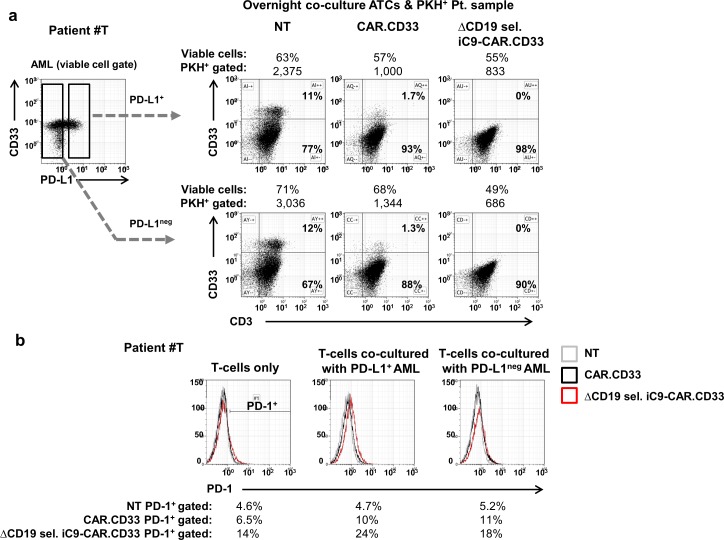
Co-culture of CAR T-cells with PD-L1^+^ AML blasts. (a) Non Transduced (NT), CAR.CD33, or ΔCD19 sel. iC9-CAR.CD33 activated T-cells (ATCs) generated from a healthy donor were co-cultured overnight with PD-L1^+^ *(top panels)* or PD-L1^neg^ *(bottom panels)* blasts collected from a patient with acute myeloid leukemia (AML), (patient#T). AML blasts were sorted by flow cytometry for PD-L1 expression, and labeled with the PKH-26 dye prior to co-culture. After overnight incubation 20,000 events were acquired by flow cytometry after gating on the PKH^+^ population and the reduction of CD33^+^ AML cells, as compared with the retained CD3^+^ cells is shown in the dot-plots. (b) ATCs co-cultured as per 5a were stained with a PD-1 monoclonal antibody, and PD-1 expression on the PKH^neg^/CD3^+^ population was analyzed by flow cytometry.

Next, we sought to investigate whether patient samples expressing the programmed cell death ligand 1 (PD-L1) would prevent cellular killing from CAR redirected ATCs. Therefore, in a pilot experiment, we sorted PD-L1^+^ or PD-L1^neg^ patient blasts by FACS (patient#T), for co-culture assay with NT, CAR.CD33, or ΔCD19 sel. iC9-CAR.CD33 ATCs. We observed that CAR.CD33, or ΔCD19 sel. iC9-CAR.CD33 ATCs efficiently killed PD-L1^neg^ targets, as well as PD-L1^+^ targets, (“Fig 5A”). The percentage of programmed cell death 1 (PD-1) expression on T-cells co-cultured with PD-L1^+^ AML blasts were 10% (CAR.CD33), or 24% (ΔCD19 sel. iC9-CAR.CD33), while the percentage of PD-1 expression on T-cells co-cultured with PD-L1^neg^ AML blasts were 11% (CAR.CD33), or 18% (ΔCD19 sel. iC9-CAR.CD33), (“Fig 5B”).

### Impact of CAR redirected ATCs on hematopoietic colony formation

**Fig 6:**
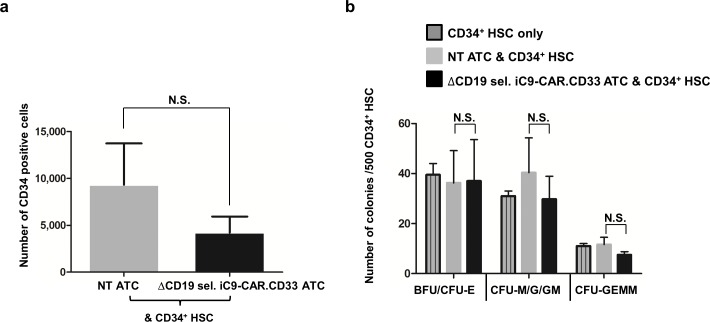
ΔCD19 sel. iC9-CAR.CD33 ATCs demonstrate limited toxicity against normal hematopoietic stem cells. (a) Human CD34^+^-selected hematopoietic stem cells (CD34^+^ HSC) from healthy volunteer donors were co-cultured overnight with or without non transduced (NT) or ΔCD19 sel. iC9-CAR.CD33 activated T-cells (ATCs), generated from healthy donors at an effector:target (E:T) ratio of 4:1. Afterwards, CD34^+^ HSC were isolated with hematopoietic lineage depletion kit, and enumerated by flow cytometry; (mean±SEM, N:4, from duplicate experiments using ATCs from 2 different healthy donors). (b) 500 CD34^+^lyneage^-^ HSC, from each respective condition outlined above, were plated on cytokine conditioned semisolid medium, *(MethoCult H4434)*. After 14 days, hematopoietic colony formation was scored using standard morphologic criteria; (mean±SEM, N:4, from duplicate experiments using ATCs from 2 different healthy donors). CFU: Colony-forming unit: BFU-E: burst-forming unit-erythroid: CFU-M/G/GM: CFU-macrophage/granulocyte/granulocyte, macrophage; CFU-GEMM: CFU-granulocyte, erythroid, macrophage, megakaryocyte; SEM: standard error of the mean; NS: not statistically significant.

In order to address whether suicide gene modified anti-CD33-CAR-redirected-ATCs would impair hematopoietic colonies formation, we used a standard *in vitro* hematopoietic colony formations assay. We co-cultured NT or ΔCD19 sel. iC9-CAR.CD33 ATCs with CD34^+^ enriched human hematopoietic stem cells (HSC). We seeded 400,000 effector cells and 100,000 CD34^+^ HSC (effector:target = 4:1). After overnight co-culture, we sorted CD34^+^ HSC by lineage negative depletion, and enumerated the remaining number of CD34^+^ HSC by flow cytometry. We found that the number of CD34^+^ HSC decreased from 9,318±4,413 when using NT as effectors, to 4,213±1,719 when using ΔCD19 sel. iC9-CAR.CD33: as effectors; *P* = 0.41 (N = 4 experiments in duplicate using ATCs generated from 2 healthy donors), (“Fig 6A”).

After co-culture, we plated the same number of residual lineage negative depleted CD34^+^ HSC in cytokine conditioned semisolid medium *in vitro*. We found that HSC co-cultured with ΔCD19 sel. iC9-CAR.CD33 ATCs retained the capability to proliferate and differentiate in cytokine conditioned semisolid medium. In fact, we scored that erythroid burst/colony forming units were 36±13; 37±17, when using NT, or ΔCD19 sel. iC9-CAR.CD33 effectors, respectively, granulocyte-macrophage colony forming units were 40±14 vs. 30±9.2, and granulocyte-erythroid-macrophage-megakaryocyte colony forming units were 12±2.9 vs. 7.5±1.3, respectively, (“Fig 6B”).

### Conditional elimination of ΔCD19 sel. iC9-CAR.CD33 ATCs

Finally, we sought to quantify the degree of CAR redirected ATCs elimination after activation of the iC9 suicide gene for safety. We performed an *in vitro* killing assay with NT, CAR.CD33 or ΔCD19 sel. iC9-CAR.CD33 treated or untreated overnight with a small molecule CID (AP20187), that activated the iC9 safety switch with resulting apoptosis of gene modified cells. After overnight exposure to CID [10 nM] *in vitro*, the killing effect on NT ATCs or CAR.CD33 ATCs was negligible with only 1.7±0.6%, and 0.3±0.7% cell elimination, respectively, whereas the elimination of iC9 suicide gene modified CAR T-cells was 76.4±2.0%, (*P*<0.001).

We tested the CID at a concentration of 14.2 ng/mL [10 nM] because this concentration resulted in maximal killing (plateau) *in vitro* [30]. *In vivo* the peak concentration assessed in healthy volunteer donors after the therapeutic dose of 0.5 mg/kg was 626 ng/mL declining after 2–4 hours.[31]

**Fig 7:**
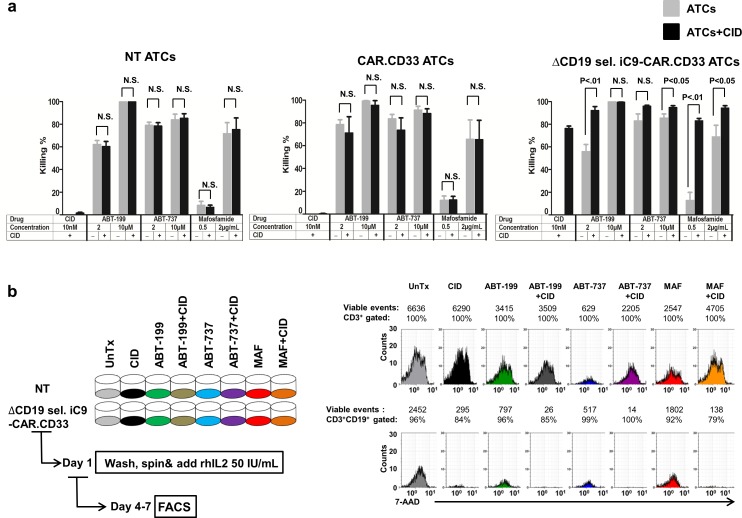
Pharmacologic elimination of ΔCD19 sel. iC9-CAR.CD33 ATCs. (a) NT, CAR.CD33, or ΔCD19 sel. iC9-CAR.CD33 ATCs generated from healthy donors were treated overnight with ABT-199 or ABT-737 [both at 2 or 10μM], or mafosfamide [0.5 or 2 μg/mL] with or without the CID [10 nM], and thereafter washed by centrifugation. On day 1, cells were harvested, stained with Annexin V/7-AAD and analyzed by flow cytometry. Ten to twenty thousand total events were acquired for each sample (the same number of events was acquired within each experiment). Average percentage (±SEM) of cell killing is represented in the histogram graph. Conditions without application of the CID are shown as shaded bars, while conditions involving the application of the CID are shown as black bars; (N:3–15 experiments using ATCs from 3–8 different healthy donors). (b) Cells treated overnight with ABT-199 or ABT-737 [both at 2 μM], or mafosfamide [0.5 μg/mL] (with or without CID [10 nM]) were harvested, washed by centrifugation and cultured for additional 4–7 days in the presence of culture medium supplemented with rhIL2 50 I.U./mL. Outline of the experimental plan, *(left)*, and histogram data from a representative experiment displaying residual viable cells is shown, *(right)*. UnTx: untreated; SEM: standard error of the mean; CID: chemical inducer of dimerization to activate the iC9 suicide gene; rhIL2: recombinant human interleukin-2; ABT-199: BCL-2 inhibitor; ABT-737: Pan-BCL inhibitor; MAF: Mafosfamide; NS: not statistically significant.

In our current *in vitro* experiments, we observed that iC9 resistant cells maintained CAR expression (80.9±2.1%), albeit at a dimmer intensity (“Figure F in S1 File”), and, considering the need to completely eliminate the CAR T-cells in case of SAEs, we aimed here to develop a strategy that completely eliminated iC9-CAR^+^ T-cells. Since we have previously reported that even increasing the concentration of the CID in both *in vitro* [30] or *in vivo* [25] settings would not result in superior cell elimination, we investigated whether the addition of a second pharmacologic agent would exert a synergistic/additive elimination effect. Based on our previous observation that iC9 resistant cells have higher expression of the anti-apoptotic molecule BCL-2 [25], we decided to investigate the killing effect of the BCL-2 inhibitor ABT-199 [2 or 10 μM: 1.74/8.7 μg/mL respectively], the pan-BCL inhibitor ABT-737 [2 or 10 μM: 1.6/8 μg/mL respectively], or mafosfamide [0.5 or 2 μg/mL], alone or in combination with the CID. We performed overnight co-culture experiments using NT, CAR.CD33 or ΔCD19 sel. iC9-CAR.CD33 ATCs, treated or untreated with the afore mentioned compounds, followed by apoptosis assay; (N:3–15 experiments using ATCs generated from 3–8 different healthy donors). ABT-199, resulted in the following percentages of NT ATCs elimination when used at 2 μM or 10 μM, respectively: (62.3±3.3%; 99.8±0.1%), or CAR.CD33 ATCs elimination: (78.6±4.2%; 99.3±0.5%), or ΔCD19 sel. iC9-CAR.CD33 ATCs elimination: (55.9±6.2%; 99.8±0.1%). Co-administration of the CID [10 nM] with ABT-199 affected only ΔCD19 sel. iC9-CAR.CD33 ATCs elimination with an additive effect: (92.1±3.5%, *P* < .01); 99.5±0.5%), when used at 2 μM or 10 μM, respectively. ABT-737, resulted in the following percentage of NT ATCs elimination when used at 2 μM or 10 μM, respectively: (79.2±2.6%; 84.0±4.8%), or CAR.CD33 ATCs elimination: (83.7±3.8%; 91.3±3.2%), or ΔCD19 sel. iC9-CAR.CD33 ATCs elimination: (83.0±6.2%; 85.5±3.5%). Again, co-administration of the CID [10 nM] with ABT-737 resulted in an additive effect on ΔCD19 sel. iC9-CAR.CD33 ATCs elimination of 96.0±0.8%, and 94.9±1.4% (*P* < .05), when used at 2 μM or 10 μM, respectively. Mafosfamide used at 0.5 or 2 μg/mL resulted in the elimination of 8.5±3.5%; 71.8±9.6% NT ATCs, 12.1±3.8%; 65.7±16.8% CAR.CD33 ATCs, or 12.9±7.0%; 69.0±10.2% ΔCD19 sel. iC9-CAR.CD33 ATCs, and, when co-administered with the CID [10 nM], those values increased to 83.1±2.0% (*P* < .01), or 94.3±2.1% (*P* < .05), when treating ΔCD19 sel. iC9-CAR.CD33 ATCs, when used at used at 0.5 or 2 μg/mL, respectively (“Fig 7A”). Afterwards, cells from each condition were re-plated with fresh T-cell culture medium in the presence of 50 I.U. /mL recombinant human IL-2 (rh-IL2), and cultured for 4–7 more days, followed by 7-AAD staining to assess their viability. A schematic representation of the experimental procedure is depicted in “Fig 7B” (left panel). At this second point analysis, the combination of either ABT-199 or ABT-737 at 2 μM together with the CID, resulted in ≥98% suicide gene modified CAR ATCs cells elimination (*P* < .01; *P* < .05, respectively), increasing to 100% when used at 10 μM. Mafosfamide at 0.5 μg/mL (with CID [10 nM]), resulted in 97.4% elimination of suicide gene modified CAR ATCs (*P* < .01), and 99% when applied at 2 μg/mL. Representative histograms displaying the number of viable cells (7-AAD^neg^) are shown in “Fig 7B” (right panels), and a summary of the results is represented in the “Figure G in S1 File”.

## Discussion

Chimeric antigen receptor redirected T-cells are an emerging powerful tool for the treatment of patients with cancer. Particularly encouraging results were demonstrated when infused CAR T-cells redirected against the CD19 antigen in patients with relapsed/refractory acute lymphoblastic leukemia resulted in a high-rate of long-lasting complete remissions. Interestingly, CAR T-cells were able to effectively expand *in vivo* in these patients, and disease responses were observed as early as 7 days after CAR T-cell infusion [4].

In contrast, the clinical experience with CAR T-cells in patients with AML is still extremely limited. Only the results from two small clinical trials have been published to date. In one trial, patients received infusion of autologous activated T-cells engineered to express a second generation CAR targeting the Lewis Y (LeY or CD174) difucosylated carbohydrate antigen. LeY is a blood group antigen shown to be present also on hematopoietic progenitors (CD34^+^ cells), on AML blasts and epithelial cancers [13]. Four patients were treated on this clinical trial, three with minimal residual disease consisting of persisting cytogenetic alterations, and one with active disease. The patients received a median number of 1.1x10^9^ (range 5x10^8^-1.3x10^9^) T-cells with a 14–38% CAR expression, as assessed by flow cytometry. In these patients, T-cells were able to expand *in vivo* (~1,200 transgene copies/1,000 cells in the peripheral blood), persisted up to 10 months, and efficiently homed to bone marrow and sites of *leukemia cutis*. Regarding clinical activity, one patient was able to achieve cytogenetic remission, another experienced a reduction in peripheral blast count, and one patient had a protracted remission of disease. No serious adverse events were observed despite the increased production of interferon-gamma detected in two patients. It is possible that the limited clinical efficacy could be secondary to the low intensity of expression of the LeY antigen on AML blasts, which is more strongly expressed on tissues of epithelial origin.

A second clinical trial infused autologous T-cells bearing a second generation CAR targeting the CD33 antigen in a patient with refractory AML and the presence of active disease. This patient received 1.12x10^9^ T-cells with a 38% CAR expression. This patient experienced mild cytokine release syndrome and a marked decrease in bone marrow blasts (from >50% to <6%) two weeks after infusion; however, this was followed by florid progression nine weeks after the T-cell infusion. Interestingly, CAR T-cells were still present in the patient’s peripheral blood and were able to kill *in vitro* leukemia cells previously isolated from the same patient. Antigen down-regulation was not the implicated mechanism of tumor evasion, since the patient blasts still highly expressed CD33 at progression. The authors postulated that leukemia cells might have adopted other immune-evasion strategies enabling them to escape the killing by CAR.CD33 ATCs.

Additionally, considering the transient but significant tumor response, the authors suggested that CAR.CD33 T-cells could be used as a ‘bridge’ to an allo-HSCT [14], thus rescuing possible off-target myelotoxic effects of the CAR.CD33 T-cells with the HSCT. Although we did not observe a significant elimination of hematopoietic stem cells *in vitro*, CAR T-cells targeting CD33 could exacerbate anemia and thrombocytopenia in virtue of CD33 expression on these hematopoietic progenitors, as previously observed in preclinical models *in vitro* [6–8], *or in vivo* [9]. Although our *in vitro* data conflicts with those previous reports, a direct comparison of the results from different studies from different centers is difficult because of different details in the performance of these assays, stem cell source, effector to target ratio, and incubation time. It is also possible that CD33 negative cells may reacquire CD33 expression during hematopoietic development, as demonstrated for CD123 negative hematopoietic progenitors [5]. In fact, conflicting results have been reported between different centers regarding other targeted antigens. CAR.CD123, for example, whereas some authors demonstrated no impact, of CAR.CD123 on myeloablation when compared with untransduced T-cells or controls [9, 11], others have shown the ability to cause myeloablation in xenogeneic HSCT mice models [5, 12], and have proposed its use as conditioning to an allo-HSCT.

Clinical trials will be helpful in understanding the impact of targeting CD33 with CAR ATCs on myelosuppression. In the only CAR.CD33 ATCs clinical case reported in literature, the patient was already neutropenic (0.4–1.2 x10^9^ cells/L) at the moment of CAR T-cell infusion, and fluctuation of the counts could have been potentially related to cytokine release syndrome. The patient from this report only had a transient marked decrease of tri-lineage blood cells within 2 weeks of the CAR.CD33 ATCs infusion. The subsequent neutrophil recovery and the active bone marrow hyperplasia suggested that irreversible myelosuppression might not have resulted from CAR.CD33 T-cell treatment. A normal myeloid compartment with low CD33 expression may survive and then compensate for the loss of a compartment with high CD33 expression in the later stage of CAR.CD33 ATCs infusion. The authors concluded that the observations from this patient should undoubtedly make us rethink that irreversible myelosuppression and neutrophil deficiency may not be an unsurpassable hurdle for CAR.CD33 T-cell therapy. Additionally, only a transient hyperbilirubinemia within 2 weeks of CAR.CD33 T-cell infusion was observed in this patient. This trial is still ongoing, and data from a larger group of patients is needed. Of note, the cytotoxic activity was modest *in vitro* [14]. In contrast, in our *in vitro* data the cytotoxic activity was robust; this may relate to the presence of additional co-stimulatory domains in our CAR (3^rd^ generation construct), the affinity/avidity profile of the *scFv*, or the T-cell generation protocol. For example, unlike our short-term culture *in vitro*, T-cells cultured long-term may exhibit a phenotype consistent with an exhausted state when using a protocol that expands the cells *in vitro* over a protracted period of time. Additionally, our CAR.CD33 was able to lyse CD33^+^ primary AML samples *in vitro* even when expressing CD33 at low levels, as previously reported by Pizzitola *et al*. [9], a finding attributed to the high affinity of the scFv used in the CAR, as in ours.

One additional immune-evasion strategy to take into account is correlated with the expression and/or secretion of an immune checkpoint molecule, such as the PD-L1 molecule by AML cells [32] [33] [34]. In our *in vitro* co-culture experiments we observed cytotoxic activity from CAR T-cells challenged with either PD-L1^neg^ or PD-L1^+^ AML blasts, although it is possible that in the *in vivo milieu*, this mechanism could contribute to increased T-cell apoptosis for binding the respective receptor on T-cells, as PD-1^+^ CD8 T-cells were found to increase in AML metastatic site *in vivo* [35].

Additionally, it is possible that anti-leukemia treatments could further increase immune-evasion mechanisms adopted by tumor cells, and therefore PD-1/PD-L1 blockade could result in an enhanced therapeutic response. In fact, *in vitro* application of the CD3/CD33 engaging bispecific antibody resulted in up-regulation of PD-L1 expression on primary AML cells, up-regulation of PD-1 expression on T-cells, with reversal of the killing inhibition through PD-1/PD-L1 blockade [36]. The impact on the removal of CD33^+^ cells, including the potential for myelosuppression, or immune-defects from targeting CD33^+^ memory T-cells will need to be investigated.

It would be meaningful to incorporate CAR T-cells as a mean of conditioning for an allo-HSCT, which could reduce the toxic effects of high-dose chemotherapy while exerting a potential positive impact on the quality of life [37]. Additionally, it could enable older patients, or patients with comorbidities to become eligible for an allo-HSCT.

Targeting a myeloid associated antigen could result in myelosuppression, but if used in conjunction with allo-HSCT, it could favorably impact the risk of disease relapse considering the clonal complexity of AML, where relapse can manifest due to the persistence of a leukemic clone, or by the acquisition of additional mutations in pre-leukemic stem cells which are contained within stem cells with a normal phenotype [38] [39]. In this scenario, the application of a suicide gene is essential to completely eliminate the infused T-cells before transplant, therefore reducing the potential risk of engraftment failure from targeting a proportion of the infused CD34^+^ hematopoietic stem cells that will express CD33. Another group has proposed the use of CAR T-cells bearing a ‘biodegradable’, transiently expressed *mRNA CAR* construct targeting CD33, delivered by the means of electroporation. They demonstrated the ability to control AML tumor growth *in vitro* and *in vivo* in mice, as well as the ability to induce myelosuppression in a humanized HSCT mice model [12]. It will need to be investigated in a clinical trial whether the limited persistence of *mRNA CAR* T-cells would exert a sufficient impact on the leukemia tumor burden. The authors are proposing to perform repeated administration of *mRNA CAR* T-cells to obviate to the limited persistence of the CAR expression. Another potential side effect of repeated administration of CAR T-cells has been observed in a previous clinical study where patients receiving T-cells electroporated with anti-mesothelin *mRNA CAR* bearing a mouse anti-human *scFv* experienced anaphylaxis from the development of anti-mouse antibodies [40].

Considering that the activation of the iC9 may result in sparing of a proportion of the CAR T-cells, we have investigated the incorporation of additional pharmacologic agents in order to render a more complete elimination of the gene modified cells. Since in our current *in vitro* experiments we observed that iC9 resistant cells maintained CAR expression, albeit at a dimmer intensity, we are planning to investigate in a dedicated project, the function of iC9-CAR^+^ resistant cells (cytotoxicity, cytokine profile after re-stimulation with antigen specific targets). In our current investigation we focused on obtaining 100% iC9-CAR^+^ cell elimination, in order to avoid the risk of graft failure. Our previous work in both animal models [25] and patients [26] has consistently demonstrated that higher dose or repeated administration of the dimerizer to activate the safety switch did not result in complete elimination of iC9 transduced cells. Our earlier studies also suggested that high levels of transcription of the *iC9* transgene caused by TCR activation in alloreactive T-cells explain the selective elimination of these cells by the dimerizer in patients who have clinical evidence of acute graft versus host disease [21]. Chang *et al*. [41] proposed that it is plausible that a dynamic process such as TCR stimulation rather than static repression mechanisms, such as epigenetic marks, affects gene promoters proximal to vector insertion sites promoting their transition from transcriptionally paused promoters to released promoters. TCR stimulation may thus indirectly modulate iC9 expression by alleviating transcriptional repression from nearby host promoters and promote iC9 mRNA expression with resulting selection of a subset of T-cells with proviral integration sites that favor low gene expression. Mutations in the iC9 molecules were not found to exert significant contribution to the dimerizer resistance [41]. Another possible mechanism of resistance is the selection of cells with an imbalance favoring anti-apoptotic factors over pro-apoptotic factors. In fact, we have previously demonstrated that iC9 resistant cells have higher expression of the anti-apoptotic molecule BCL-2 [25]. Therefore, in addition to searching for additional pharmacologic agents able to grant complete elimination of iC9 T-cells, we decided to investigate the impact of agents able to inhibit anti-apoptotic factors. We observed an additive effect on T-cell elimination when combining the BCL-2 inhibitor ABT-199, with the dimerizer to activate the iC9 suicide gene, at a concentration comparable to bioequivalent doses obtained in patients at currently investigated therapeutic doses [42]. Clinical safety data from the performed trial showed that ABT-199 had modest impact on myelosuppression, except for grade 3–4 neutropenia in 41% of patients [42]. ABT-199 is an agent currently being investigated for the treatment of AML [27], and it would be interesting to use targeted therapy sequentially with CAR T-cells, in order to exert a synergistic effect on resistant leukemia clones [43]. We also added, at a similar concentration, the pan-BCL inhibitor ABT-737 which resulted in a potent lymphotoxic effect as well [29]. These drugs could also result in the elimination of non-gene modified T-cells, therefore alternative strategies to enhance the elimination of only iC9 suicide gene modified T-cells are needed, and are currently under investigation in our lab. It remains to be demonstrated whether BCL-2 inhibitors would exert a superior killing effect (albeit not completely sparing normal T-cells), on iC9-cells resistant to AP1903, in virtue of their higher BCL-2 expression.

Additionally, although cells can develop an escape mechanism by up-regulating alternative anti-apoptotic molecule, this is unlikely to develop during short-term treatment, and furthermore, the killing effect observed with this agent when combined with the dimerizer was profound.

It is unlikely that BCL-2 mediates its anti-apoptotic effects through direct interaction with iC9. Other possible negative checkpoints in the Caspase 9 activation pathway involve the interaction between the BIR-3 domain of XIAP with Caspase9 and Caspase3, [44] as well as the interaction of BCL-XL with APAF-1, [45] although the latter is bypassed by inducing iC9 with exogenous administration of the dimerizer. We also found that co-administration of mafosfamide [28] was able to exert a more profound elimination of suicide gene modified T-cells, as compared to the CID alone, a relevant finding since the bio-equivalent dose resulting from the administration of intermediate-doses [1.5–2,000 mg/mq] of cyclophosphamide could be incorporated as part of a reduced intensity preparative chemotherapeutic regimen [28].

In summary, we show here the pre-clinical activity of a third generation CAR targeting the CD33 antigen for AML. We co-expressed an iC9 suicide gene for safety, and were able to completely eliminate suicide gene modified T-cells through the administration of a non-therapeutic dimerizer agent and an additional targeted agent.

Although we are aware and have planned to prove the efficacy and safety of our anti-CD33.CAR transduced ATCs in a murine xenogenic model, our results indicate that this strategy could be investigated for the safety of CAR T-cells applications. Additionally, anti-CD33 redirected CAR T-cells could be used as a ‘bridge” therapy for patients coming to an allo-HSCT, as anti-leukemia activity from infusing CAR.CD33 T-cells has been demonstrated in an ongoing clinical trial. [14] Finally, albeit never performed in the clinical setting, our future plan is to investigate the utility of iC9-CAR.CD33 T-cells as part of the conditioning therapy for an allo-HSCT for AML, together with other myelosuppressive agents, whilst the activation of the iC9 suicide gene would grant elimination of the infused gene modified T-cells prior to stem cell infusion to reduce the risk of engraftment failure as CD33 is also expressed in a proportion of the donor stem cell graft.

## Materials and Methods

### Tumor cell lines and patient samples

All cell lines were obtained from the American Type Culture Collection *(ATCC; Manassas*, *VA)*. For cytotoxicity assays we employed the CD33^+^ biphenotypic B-myelomonocytic leukemia derived human cell line MV4-11. Peripheral blood or bone marrow samples were collected from healthy donors and patients with AML after written informed consent in accordance with the Declaration of Helsinki. The Institutional Review Board of the University of Alabama for Human Use at Birmingham (Birmingham, AL) approved this study (IRB# F140702002). MV4-11 and 293T cells were maintained in culture with IMDM medium *(Thermo Fisher Scientific*: *Waltham*, *MA)* containing 10% fetal bovine serum (FBS) *(GE Healthcare Life Sciences*: *Logan*, *UT)*, and 2mM L-glutamine *(Thermo Fisher Scientific)*. Peripheral blood or bone marrow cells were maintained in culture with T-cell medium composed of 45% RPMI 1640 *(GE Healthcare Life Sciences)*, 45% Click’s media *(Irvine Scientific*, *Santa Ana*, *CA)* supplemented with 10% FBS, and 2 mM L-glutamine. MV4-11, MV4-11-eGFP, and 293T cell lines tested negative for mycoplasma contamination using the Mycoplasma PCR ELISA Kit *(Roche Diagnostics GmbH; Mannheim*, *Germany)*. We confirmed MV4-11 and MV4-11-eGFP cell lines identity using short tandem repeat (STR) DNA profiling for CSF1PO, D10S1248, D12S391, D13S317, D16S539, D18S51, D19S433, D1S1656, D21S11, D22S1045, D2S1338, D2S441, D3S1358, D5S818, D7S820, D8S1179, DYS391, FGA, Penta D, Penta E, TH01, TPOX, vWA, and Amelogenin, *(UAB Heflin Center for Human Genetics with AmpFlSTR system (Applied Biosystems; Foster City*, *CA)*.

### Retroviral constructs

#### CD33 chimeric antigen receptor (CAR.CD33)

In order to generate the *CAR*.*CD33* construct, we synthesized a humanized anti-CD33 *scFv*, retrieved from Patent *US 8*,*747*,*851*, using G-block technique *(IDT Technologies*, *Coralville*, *Iowa)*, inserting a *CD8 alpha protein leader* sequence upstream. *T*he synthesized construct was then ligated into *SFG-X-CD28-OX40ζ* retroviral vector (kindly provided by Dr. J. Maher *(King’s College of London))* [46], using restriction nucleases technique.

#### Inducible Caspase9 vector with truncated (non-functional) CD19 and CD33 chimeric antigen receptor (ΔCD19 sel. iC9-CAR.CD33)

For the *iC9-*Δ*CD19-CAR*.*CD33* vector, we generated a construct encoding the iC9 suicide gene and the ΔCD19 selectable marker (patent US9089520), as well as the CAR.CD33 vector co-expressed through the incorporation of the *2A sequence* from Thosea *Asigna* virus using restriction nucleases technique.

#### Luciferase vectors

The generation of retrovirus vectors encoding the fusion protein eGFP-ffLuc has previously been described. [47] After transduction, eGFP positive cells were sorted by FACS, *(FACS Aria II*, *BD Biosciences*, *San Jose*, *CA)*. To confirm luciferase transgene function, 2.5x10^4^ cells in 50 μL of medium were lysed with 50 μL of D-luciferin according to the manufacturer’s instructions *(Promega*, *Madison*, *WI)*, and the bioluminescence signal was read at the luminometer *(Synergy H1 Hybrid*, *BioTek*, *Winooski*, *VT)*. The eGFP-ffLuc vector was used to transduce the MV4-11 cell line or ATCs; eGFP expression was assessed by FACS analysis *(FACS Calibur*, *Canto II*, *BD)*, whereas expression of ffLuc was detected using D-luciferin. The integrity of cloning for all constructs used in this manuscript was confirmed by Sanger sequencing performed by the Heflin Center for Human Genetics of the University of Alabama at Birmingham, using the BigDye Terminator v3.1 Cycle Sequencing Ready Reaction kit as per the manufacturer's instructions (Applied Biosystems, Foster City, CA). The sequencing products were run following standard protocols on an Applied Biosystems 3730 Genetic Analyzer with POP-7 polymer.

#### Transduction of human ATCs

To transduce human ATCs replication incompetent retroviral supernatant was prepared by transfecting *293T* with DNA encoding our construct of interest, the *Peg-Pam-e* plasmid containing the sequence for *MoMLV gag-pol*, and the *DRF* plasmid containing the sequence for the *RD114* envelope, as previously described.[48] Supernatant harvested at 48 or 72 hours post transfection was used to transduce human ATCs. ATCs were generated using PBMCs from healthy volunteer donors or patients with CD33^+^ AML, activated with anti-CD3/CD28 antibodies *(BD Biosciences)*, and expanded with rh-IL2 50–100 I.U./mL twice weekly *(Miltenyi Biotec; San Diego*, *CA)*. For ΔCD19 sel. iC9-CAR.CD33 CAR T-cells, we sorted ΔCD19 expressed cells with magnetic conjugated anti-CD19 antibody beads through MACS column *(Miltenyi Biotec)* between 7 to 9 days after ATCs expansion.

#### Phenotype

The following monoclonal antibodies conjugated with phycoerythrin (PE), fluorescein isothiocyanate (FITC), periodin chlorophyll protein (PerCP) and/or Allophycocyanin (APC) were used: CD3, CD4, CD8, CD16, CD56, CD19, CD20, CD33, CD34, PD-L1, PD1, CD45RA/RO, and CD62L (5μL/sample) *(all from BD Biosciences)*. Expression of the CAR on ATCs was detected using Cy-5–conjugated goat anti–human IgG (1μL/sample) (H+L) Abs (*Jackson ImmunoResearch Laboratories*: *West Grove*, *PA*), which recognize the human IgG1-CH2CH3 component incorporated within the CAR. Cells were analyzed by a FACS Calibur, or FACS Canto II *(BD Biosciences)* for fluorescence signals. For each sample, a minimum of 10,000 viable events were acquired, and analyzed using the Kaluza software v.2 *(Beckman Coulter*: *Brea*, *CA)*.

#### Cytotoxicity assay

Luminescence based cytotoxicity assays was performed using the Bright-Glo Luciferase Assay System *(Promega*: *Madison*, *WI)* according to the manufacturer’s instruction. Target cells (MV4-11 AML cell line), were engineered to express the ffLuc construct, and co-cultured overnight with effector cells, as appropriate, in 200 μl using clear bottom black 96 well plates *(Corning Incorporated)*. The luminescent signal was measured Synergy H1 Hybrid analyzer *(BioTek*: *Winooski*, *VT)* and the percentage of specific lysis was calculated using the following formula: [(maximum value of labeled targets only (max)-experimental)/max * 100].

#### Co-culture assays

The co-culture assays used the MV4-11 CD33^+^ cell line as a target, after transduction with an eGFP retroviral vector, and enriched for eGFP expression by FACS sorting *(FACS Aria II*, *BD Biosciences)*. For the co-culture assays employing leukemia blasts isolated from patients, we labeled samples collected from patients with AML with the PKH-26 fluorescent dye *(Sigma-Aldrich*, *St*. *Louis MO)* to enable its detection by FACS. We challenged the targets with NT, CAR.CD33, or ΔCD19 sel. iC9-CAR.CD33 redirected ATCs, and assessed the CD33^+^eGFP^+^ or CD33^+^PKH^+^surface marker’s expression after overnight incubation. We cultured, stained and resuspended the cells in the same volumes of saline buffer. We acquired the same number of total events (10–50,000 viable and dead cells; constant for each experimental conditions) in the forward and side scatters (FS/SS) gate on FACSCalibur. In order to confirm that the marker reduction was due to cell killing and not to antigen down-regulation, we evaluated the target cell’s viability after 4 hours of co-culture, using a standard Annexin V/7-AAD apoptosis assay.

#### CD107a degranulation assay

Degranulation assay was performed as previously described. [12] Briefly, T-cells were incubated with eGFP^+^, or PKH ^+^ labelled targets at a 4:1 ratio. APC-conjugated-CD107a- antibody was added at the time of incubation. After 4 hours, cells were harvested and stained with anti-CD3 FACS antibody. For each sample, 50,000 events were acquired by FACS.

#### T-cell proliferation

ATCs were re-suspended at 2 × 10^6^/ml in 850 μl of phosphate-buffered saline and labeled with 150 μl of CFSE 10 μM (*Thermo Fisher Scientific*) for 10 minutes at 37°C. The reaction was then quenched with RPMI containing 10% FBS, and cells were washed by centrifugation. ATCs were incubated at a 1:1 ratio with irradiated target cells (40 Gy) for 5 days. Cells were then harvested, stained with anti-CH2CH3 and anti-CD3 monoclonal antibodies and analyzed by FACS.

#### Cytokines measurement

Supernatants from overnight co-culture experiments was collected and stored at minus 80 degrees Celsius until cytokines measurement determination for human IL-2, IL-4, IL-6, IL-10, TNF-α, IFN-γ, and IL-17A using the BD^TM^ Cytometric Bead Array (CBA) Human Th1/Th2/Th17 Cytokines Kit (*BD Biosciences*), in accordance with the manufacturer’s instruction. Briefly, supernatants were incubated with beads- and PE- conjugated anti-cytokine antibodies. Each serial diluted of standard protein was also incubated with those antibodies. After 3 hours of incubation, beads were washed and acquired by FACS. Two hundred forty thousand beads per test were acquired, to capture at least 300 events per each cytokine bead. To make standard curves and measure cytokines level, data were analyzed using FCAP Array^TM^ Software Version 3.0 (*BD Biosciences*). As a control, we measured the supernatant collected from ATCs only, or MV4-11, or patient’s tumor cells only.

#### *In vitro* hematopoietic colony formation assay

In order to assess the impact of NT versus suicide gene modified CAR ATCs on the elimination of normal hematopoietic progenitors or HSCs, CD34^+^ cells isolated from the peripheral blood of healthy donors mobilized with G-CSF were purchased from *Hemacare (Van Nuys*, *CA)*. Cells were co-cultured overnight with ATCs (NT, or suicide gene modified CAR.CD33 redirected ATCs; effector:target = 4:1). Afterwards cells underwent negative lineage depletion *(*Human Progenitor Cell Enrichment Kit with Platelet Depletion, *Stem Cell Technologies*: *Vancouver*, *BC*) and were resuspended in Iscove’s modified Dulbecco’s medium *(IMDM*, *Thermo Fisher Scientific)* and mixed with methylcellulose-based semisolid medium (*MethoCult H4434*, *Stem Cell Technologies)*. Five hundred CD34^+^ cells per ml of media mixture were plated on 35-mm petri dish, and incubate at 37°C, 5%CO2 for 14 days. Colonies were then counted and scored according to standard morphological criteria using a polarized light inverted microscope.

#### Killing assay of suicide gene modified CAR T-cells

To demonstrate the ability to eliminate suicide gene modified CAR T-cells through the activation of the iC9 suicide gene we performed *in vitro* experiments involving the administration of the non-therapeutic chemical inducer of dimerization (CID) *(AP-20187*, *Clontech; Mountain View*, *CA)*, that results in iC9 dimerization and apoptosis of iC9 modified cells. NT, CAR.CD33, or ΔCD19 sel. iC9-CAR.CD33 ATCs were incubated overnight in the presence or absence of CID [10 nM] and/or with the BCL-2 inhibitor *(ABT-199 [2 or 10 μM]; ApexBio Technology*: *Houston*, *TX)*, and/or with the pan-BCL inhibitor *(ABT-737 [2 or 10 μM]; Santa Cruz Biotech*: *Dallas*, *TX)*, and/or with mafosfamide [0.5 or 2 μg/mL] *(Santa Cruz Biotech)*. Thereafter, ATCs were washed by centrifugation, and cultured for 4–7 additional days in the presence of rh-IL2 [50 I.U./mL]. After 4–7 days, the cells were harvested, and the percentage of cell killing was estimated after Annexin V/7-AAD staining *(BD Biosciences)* and FACS analysis, using the following formula: [*100%—(%Viability treated /% Viability non-treated cells)]*.

#### Statistical analysis

All *in vitro* data are presented as average± standard error of the mean (SEM). Student *t* test was used to determine the statistical significance of differences between samples, and a (two-sided) *P-*value less than 0.05 was accepted as indicating a statistically significant difference. Average± standard error of the mean (SEM) is shown/reported for all the experiments unless otherwise indicated. Correlation coefficient was calculated with the linear regression analysis. Data were analyzed and plotted using Excel-2007 *(Microsoft*: *Redmond*, *WA)*, and GraphPad Prism *(GraphPad Software*: *La Jolla*, *CA)* software.

## Supporting Information

S1 File **Table A in S1 File. CD33 expression on PBMC, ATCs, and iC9**^**+**^ **CAR.CD33 ATCs.** PBMC: peripheral blood mononuclear cells; ATCs: activated T-cells; SEM: standard error of the mean. **Figure A in S1 File. CAR.CD33 ATCs from healthy donors: expansion.** Non transduced (NT), CAR.CD33, or ΔCD19 selected (sel.) iC9-CAR.CD33 activated T-cells (ATCs) generated from 4 healthy donors were cultured in the presence of recombinant human interleukin-2 (50–100 I.U./mL) twice weekly, and counted at weekly intervals. The line graph represents mean±SEM of the cell’s fold expansion. SEM: standard error of the mean; NS: not statistically significant. **Figure B in S1 File. Apoptosis from anti-CD33 redirected CAR ATCs.** Non transduced (NT), CAR.CD33, or ΔCD19 sel. iC9-CAR.CD33 activated T-cells (ATCs) were co-cultured with the MV 4-11-CD33^+^ cell line transduced with the enhanced green fluorescent protein marker (eGFP), at an effector: target ratio of 4:1. After overnight incubation residual viable cells (Annexin V^neg^/7-AAD^neg^) were assessed by flow cytometry after gating on eGFP^+^ targets. Ten to fifty thousand viable and dead events were acquired (the same number of events was acquired within each experiment). The percentage of viable cells is reported in comparison with co-culture employing NT ATCs as effectors; (mean±SEM of 3 experiments using ATCs from 3 healthy donors). SEM: standard error of the mean. **Figure C in S1 File. CAR.CD33 ATCs from AML patients: expansion.** Non transduced (NT), CAR.CD33, or ΔCD19 sel. iC9-CAR.CD33 activated T-cells (ATCs) generated from 2 patients with acute myeloid leukemia (pts.#3 and #U), were cultured in the presence of recombinant human interleukin-2 (50–100 I.U./mL) twice weekly, and counted at weekly intervals. The line graph represents mean±SEM of the cell’s fold expansion. SEM: standard error of the mean. **Figure D in S1 File. CAR ATCs from patient#U kill CD33**^**+**^ **targets.** Non transduced (NT), CAR.CD33, or ΔCD19 sel. iC9-CAR.CD33 activated T-cells (ATCs) from patient (pt.)#U were co-cultured overnight either with the MV4-11 CD33^+^ AML cell line genetically modified to express the enhanced green fluorescent protein (eGFP) marker, *(upper panels)*, or with the autologous CD33^+^ acute myeloid leukemia sample labelled with the PKH-26 dye, *(lower panels*). Dot plots from a representative co-culture experiment displaying CD33 vs eGFP expression on eGFP^+^ gated target cells, or CD33 vs. PKH on PKH^+^ gated target cells, respectively. Ten to fifty thousand viable and dead events were acquired (the same number of events was acquired within each experiment). **Figure E in S1 File. CAR ATCs from patient#T induce apoptosis.** Non transduced (NT), CAR.CD33, or ΔCD19 selected (sel.) iC9-CAR.CD33 activated T-cells (ATCs) from patient (pt.)#T were co-cultured 4 hours either with the MV4-11 CD33^+^ acute myeloid leukemia (AML) cell line genetically modified to express the enhanced green fluorescent protein (eGFP) marker, *(left panels)*, or with the autologous CD33^+^ acute myeloid leukemia sample labelled with the PKH-26 dye, *(right panels*); the co-cultures were performed in the presence of medium *(top panels)* or autologous patient’s plasma *(bottom panels)*. Dot plots from a representative co-culture experiment displaying Annexin V vs 7-AAD expression on eGFP^+^, or CD33^+^ gated target cells, respectively. Twenty thousand viable and dead events were acquired by flow cytometry. **Figure F in S1 File. CAR expression on residual ATCs after CID.** Non transduced (NT), or ΔCD19 sel. iC9-CAR.CD33 activated T-cells (ATCs) generated from 4 healthy donors were treated overnight with CID [10nM], washed by centrifugation and CAR expression was analyzed by flow cytometry after staining with anti-CD3 and anti-CH2CH3 monoclonal antibody. Ten to twenty thousand total events were acquired for each sample (the same number of events was acquired within each experiment). Average (±SEM) of the percentage of residual CAR expressing T-cells after CID treatment (bright (brt), dim, or total CAR expression), calculated from 5 independent experiments is shown in the histogram graph, *(left)*, and a dot-plot from a representative experiment displaying CD3 vs. CAR expression (assessed with the anti-CH2CH3 antibody) is shown, *(right)*. CID: chemical inducer of dimerization to activate the iC9 suicide gene; SEM: standard error of the mean NS: not statistically significant; UnTx: untreated; Lymph: lymphocyte. **Figure G in S1 File. Pharmacologic elimination of iC9**^**+**^ **CAR.CD33 ATCs.** Non transduced (NT), or ΔCD19 sel. iC9-CAR.CD33 activated T-cells (ATCs) generated from healthy donors were treated overnight with ABT-199 or ABT-737 [both at 2 or 10 μM], or mafosfamide [0.5 or 2 μg/mL] with or without CID [10 nM]. After overnight treatment, cells were washed by centrifugation and cultured for additional 4–7 days in the presence of culture medium supplemented with rhIL2 50 I.U./mL. After additional culture, cells were harvested, stained with Annexin V/7-AAD, and viability analyzed by flow cytometry. Ten to twenty thousand total events were acquired for each sample (the same number of events was acquired within each experiment). Average percentage (±SEM) of cell killing is represented in the histogram graph. Conditions without application of the CID are shown as shaded bars, while conditions involving the application of the CID are shown as black bars; (N:3–13 experiments using ATCs from 2–5 different healthy donors). UnTx: untreated; SEM: standard error of the mean; CID: chemical inducer of dimerization to activate the iC9 suicide gene; rhIL2: recombinant human interleukin-2; ABT-199: BCL-2 inhibitor; ABT-737: Pan-BCL inhibitor; MAF: mafosfamide; NS: not statistically significant.
(PDF)
