# Correction: *In Vitro* Pre-Clinical Validation of Suicide Gene Modified Anti-CD33 Redirected Chimeric Antigen Receptor T-Cells for Acute Myeloid Leukemia

**DOI:** 10.1371/journal.pone.0172640

**Published:** 2017-02-15

**Authors:** Kentaro Minagawa, Muhammad O. Jamil, Mustafa AL-Obaidi, Larisa Pereboeva, Donna Salzman, Harry P. Erba, Lawrence S. Lamb, Ravi Bhatia, Shin Mineishi, Antonio Di Stasi

The following information is missing from the Funding section: A.D. was supported by an American Cancer Society (ACS) New faculty Career Development Grant, and by a “Walter B. Frommeyer Jr. Fellowship in investigative medicine."
