# Supplementary material for: In Vitro Pre-Clinical Validation of Suicide Gene Modified Anti-CD33 Redirected Chimeric Antigen Receptor T-Cells for Acute Myeloid Leukemia
Source: PLoS One. 2016 Dec 1;11(12):e0166891. doi: 10.1371/journal.pone.0166891 (PMC5132227; doi:10.1371/journal.pone.0166891)
Supplement: S1 File — Table A in S1 File. CD33 expression on PBMC, ATCs, and iC9+ CAR.CD33 ATCs. PBMC: peripheral blood mononuclear cells; ATCs: activated T-cells; SEM: standard error of the mean. Figure A in S1 File. CAR.CD33 ATCs from healthy donors: expansion. Non transduced (NT), CAR.CD33, or ΔCD19 selected (sel.) iC9-CAR.CD33 activated T-cells (ATCs) generated from 4 healthy donors were cultured in the presence of recombinant human interleukin-2 (50–100 I.U./mL) twice weekly, and counted at weekly intervals. The line graph represents mean±SEM of the cell’s fold expansion. SEM: standard error of the mean; NS: not statistically significant. Figure B in S1 File. Apoptosis from anti-CD33 redirected CAR ATCs. Non transduced (NT), CAR.CD33, or ΔCD19 sel. iC9-CAR.CD33 activated T-cells (ATCs) were co-cultured with the MV 4-11-CD33+ cell line transduced with the enhanced green fluorescent protein marker (eGFP), at an effector: target ratio of 4:1. After overnight incubation residual viable cells (Annexin Vneg/7-AADneg) were assessed by flow cytometry after gating on eGFP+ targets. Ten to fifty thousand viable and dead events were acquired (the same number of events was acquired within each experiment). The percentage of viable cells is reported in comparison with co-culture employing NT ATCs as effectors; (mean±SEM of 3 experiments using ATCs from 3 healthy donors). SEM: standard error of the mean. Figure C in S1 File. CAR.CD33 ATCs from AML patients: expansion. Non transduced (NT), CAR.CD33, or ΔCD19 sel. iC9-CAR.CD33 activated T-cells (ATCs) generated from 2 patients with acute myeloid leukemia (pts.#3 and #U), were cultured in the presence of recombinant human interleukin-2 (50–100 I.U./mL) twice weekly, and counted at weekly intervals. The line graph represents mean±SEM of the cell’s fold expansion. SEM: standard error of the mean. Figure D in S1 File. CAR ATCs from patient#U kill CD33+ targets. Non transduced (NT), CAR.CD33, or ΔCD19 sel. iC9-CAR.CD33 activated T-cells (ATCs) from pati [file pone.0166891.s001.pdf]

Table A

PBMC		Non transduced		ΔCD19 sel. iC9-CAR.CD33	
Donor	CD33 expression (%)	Donor	CD33 expression (%)	Donor	CD33 expression (%)
#2	10.7	#3	33.1	#3	1.6
#3	8.0	#4	37.7	#1	0.8
#4	11.5	#1	28.6	#2	1.6
#3	0.7	#2	33.1	#4	0.6
		#4	35.9		
Average	7.7	Average	33.7	Average	1.1
SEM	2.4	SEM	1.5	SEM	0.3

Figure A

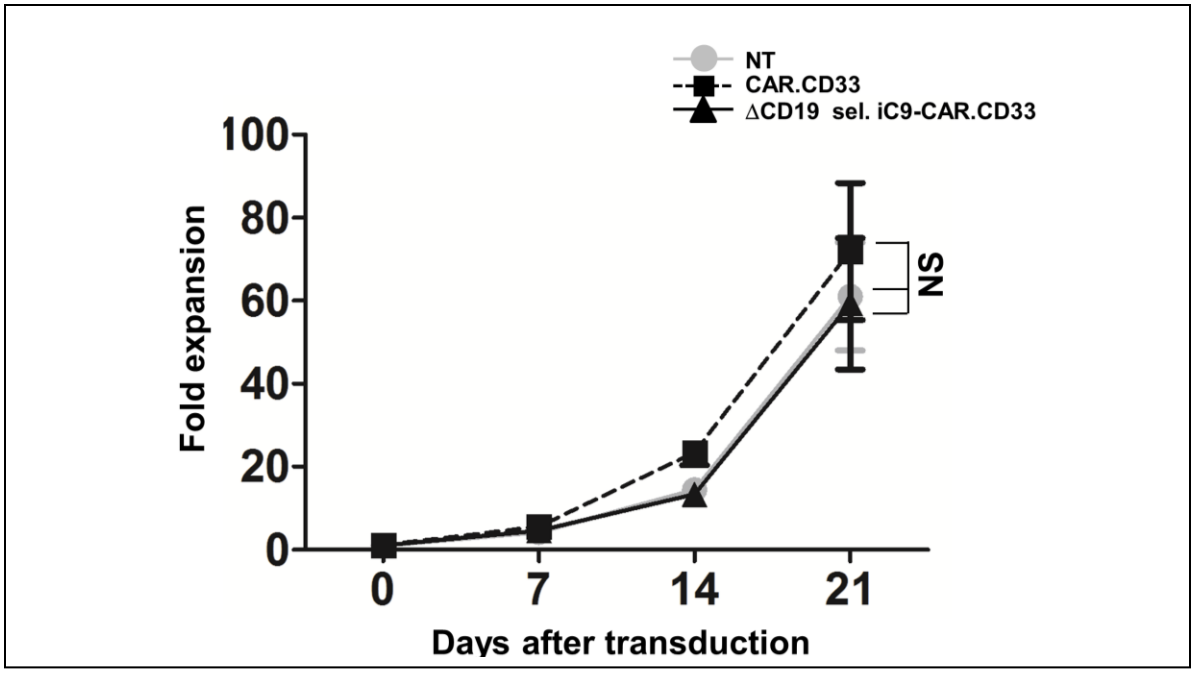
NT
CAR.CD33
ΔCD19 sel. iC9-CAR.CD33
100
80
60
40
20
0
Fold expansion
0
7
14
21
Days after transduction
NS

Figure B

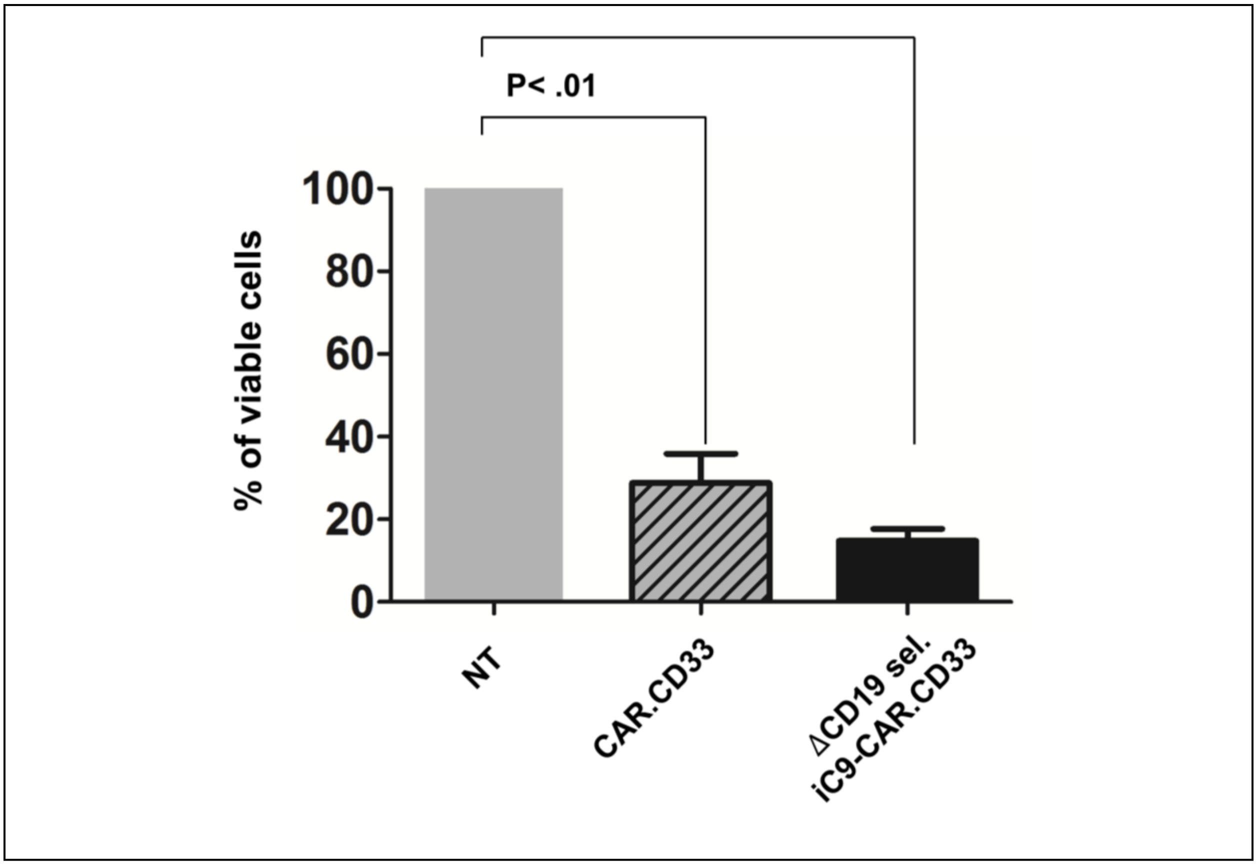

P< .01
% of viable cells
100
80
60
40
20
0
NT
CAR.CD33
ΔCD19 sel.
iC9-CAR.CD33

Figure C

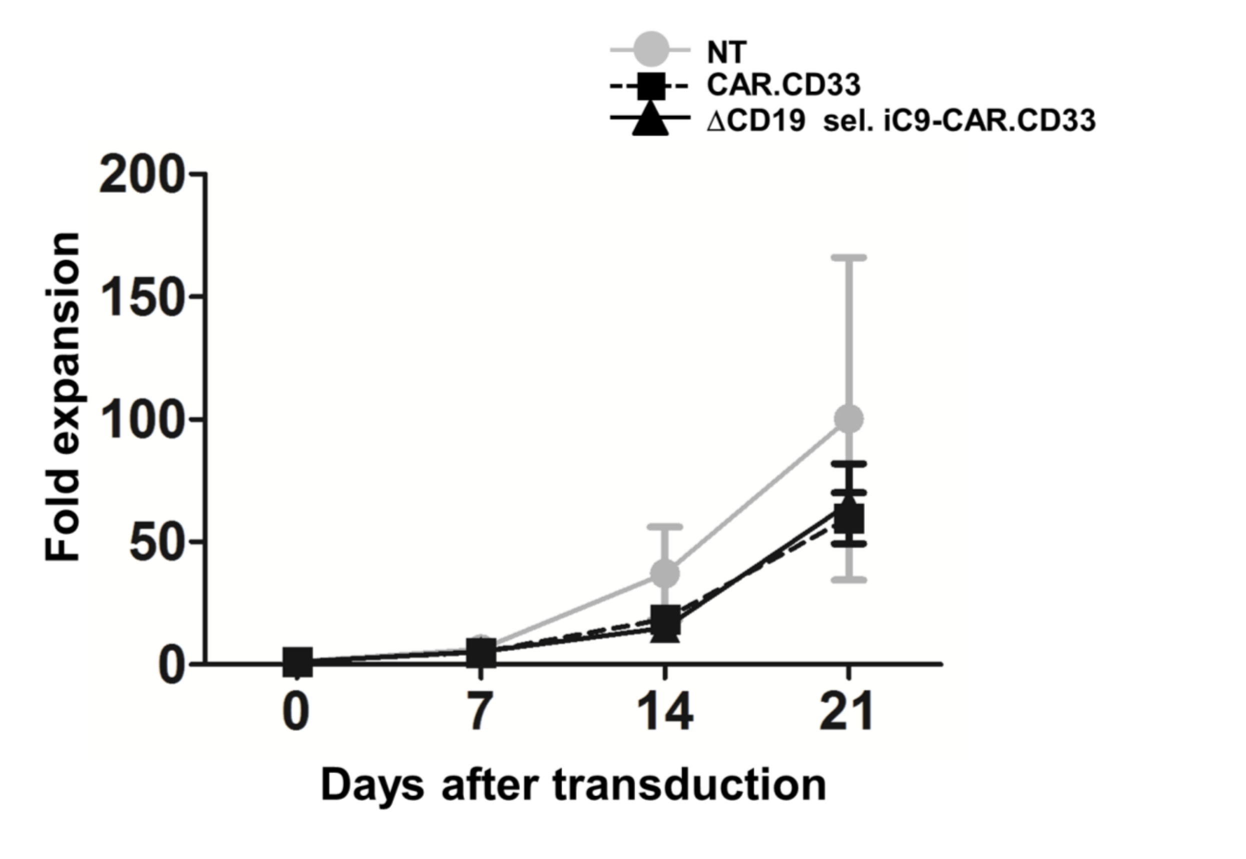
NT
CAR.CD33
ΔCD19 sel. iC9-CAR.CD33
Fold expansion
200
150
100
50
0
0
7
14
21
Days after transduction

Figure D

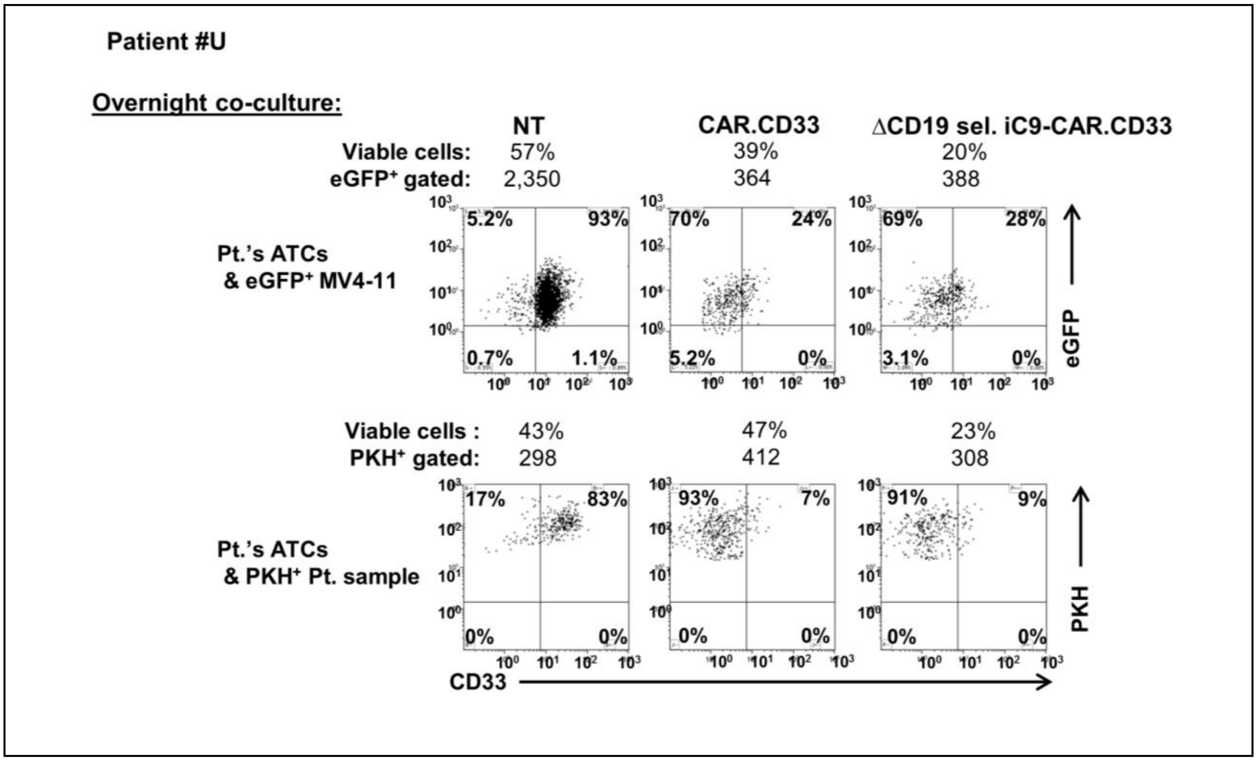
Patient #U
Overnight co-culture:
NT
CAR.CD33
∆CD19 sel. iC9-CAR.CD33
Viable cells: 57% 39% 20%
eGFP+ gated: 2,350 364 388
Pt.'s ATCs
& eGFP+ MV4-11
5.2% 93% 0.7% 1.1%
70% 24% 5.2% 0%
69% 28% 3.1% 0%
eGFP
Viable cells : 43% 47% 23%
PKH+ gated: 298 412 308
Pt.'s ATCs
& PKH+ Pt. sample
17% 83% 0% 0%
93% 7% 0% 0%
91% 9% 0% 0%
PKH
CD33

Figure E

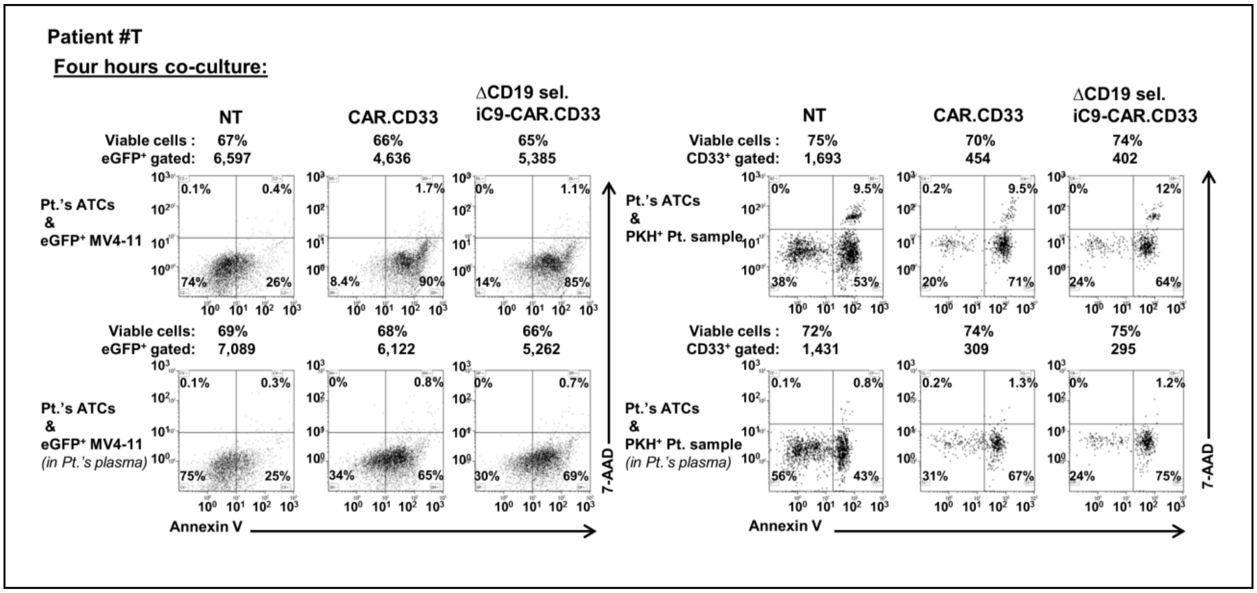

Patient #T
Four hours co-culture:
NT
CAR.CD33
ΔCD19 sel. iC9-CAR.CD33
Viable cells : 67% 66% 65%
eGFP+ gated: 6,597 4,636 5,385
Pt.'s ATCs & eGFP+ MV4-11
Viable cells: 69% 68% 66%
eGFP+ gated: 7,089 6,122 5,262
Pt.'s ATCs & eGFP+ MV4-11 (in Pt.'s plasma)
Annexin V
7-AAD
Viable cells : 75% 70% 74%
CD33+ gated: 1,693 454 402
Pt.'s ATCs & PKH+ Pt. sample
Viable cells : 72% 74% 75%
CD33+ gated: 1,431 309 295
Pt.'s ATCs & PKH+ Pt. sample (in Pt.'s plasma)

Figure F

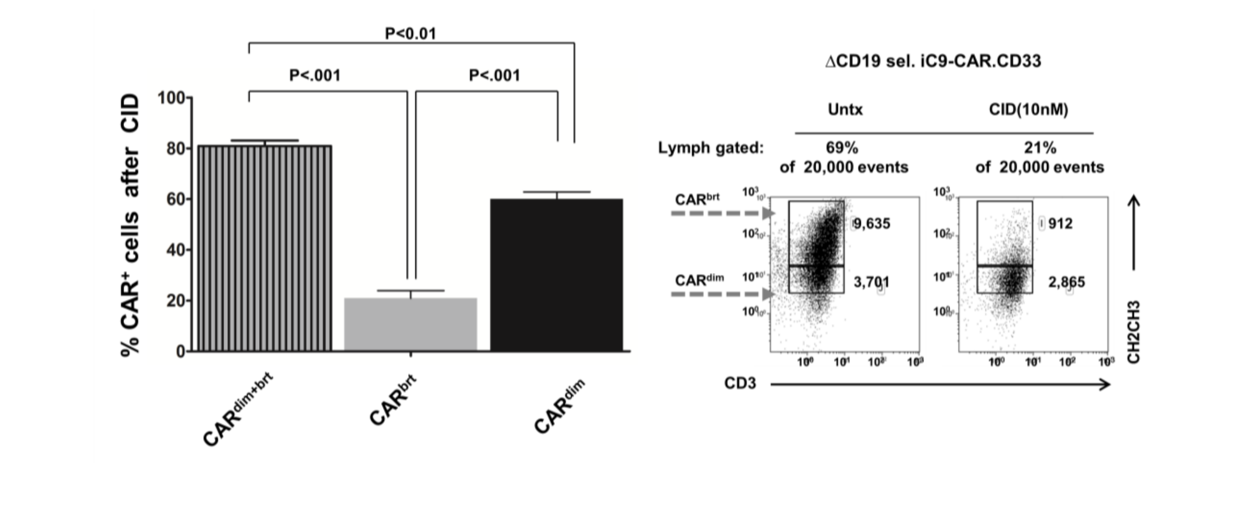

P<0.01
P<.001
P<.001
% CAR⁺ cells after CID
100
80
60
40
20
0
CARdim+brt
CARbrt
CARdim
ΔCD19 sel. iC9-CAR.CD33
Untx
CID(10nM)
Lymph gated:
69%
of 20,000 events
21%
of 20,000 events
CARbrt
CARdim
9,635
3,701
912
2,865
CD3
CH2CH3

Figure G

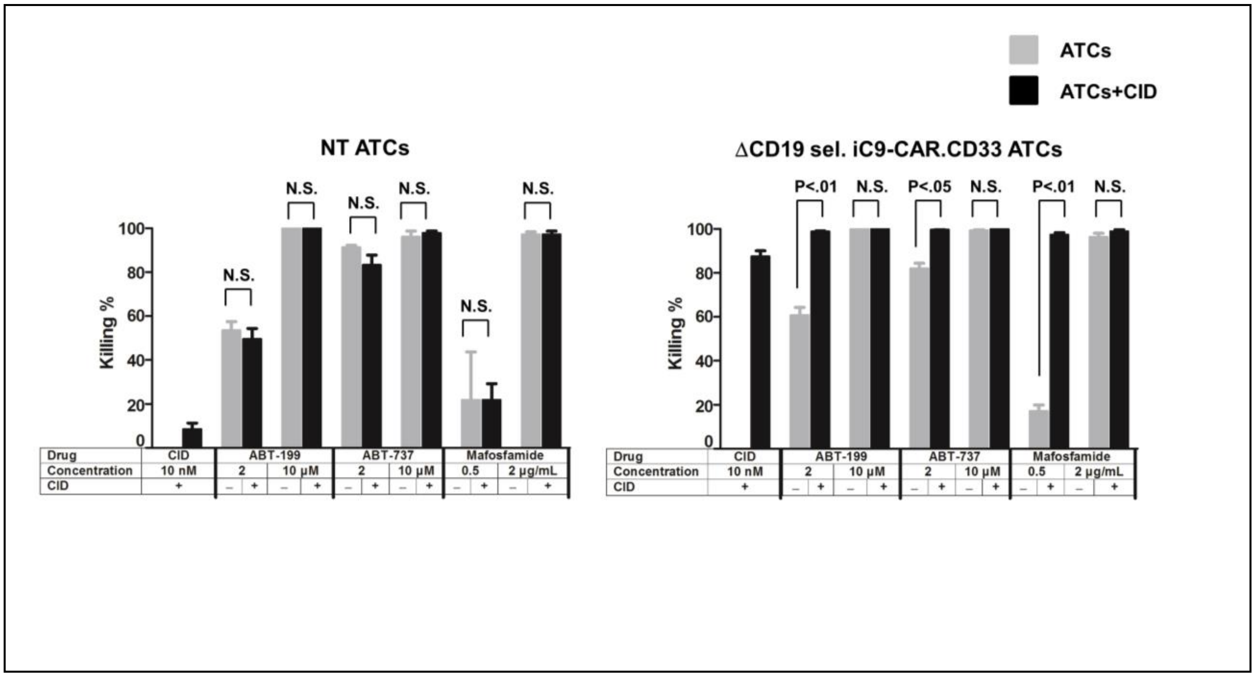

ATCs
ATCs+CID
NT ATCs
ΔCD19 sel. iC9-CAR.CD33 ATCs
Killing %
N.S.
P<.01
P<.05
Drug
Concentration
CID
ABT-199
ABT-737
Mafosfamide
10 nM
10 µM
2 µg/mL
